# Evidence for the Benefits of Melatonin in Cardiovascular Disease

**DOI:** 10.3389/fcvm.2022.888319

**Published:** 2022-06-20

**Authors:** Mohammad Tobeiha, Ameneh Jafari, Sara Fadaei, Seyed Mohammad Ali Mirazimi, Fatemeh Dashti, Atefeh Amiri, Haroon Khan, Zatollah Asemi, Russel J. Reiter, Michael R. Hamblin, Hamed Mirzaei

**Affiliations:** ^1^School of Medicine, Kashan University of Medical Sciences, Kashan, Iran; ^2^Student Research Committee, Kashan University of Medical Sciences, Kashan, Iran; ^3^Advanced Therapy Medicinal Product (ATMP) Department, Breast Cancer Research Center, Motamed Cancer Institute, ACECR, Tehran, Iran; ^4^Proteomics Research Center, Shahid Beheshti University of Medical Sciences, Tehran, Iran; ^5^Department of Internal Medicine and Endocrinology, Beheshti University of Medical Sciences, Tehran, Iran; ^6^Department of Medical Biotechnology, School of Medicine, Mashhad University of Medical Sciences, Mashhad, Iran; ^7^Department of Pharmacy, Abdul Wali Khan University, Mardan, Pakistan; ^8^Research Center for Biochemistry and Nutrition in Metabolic Diseases, Institute for Basic Sciences, Kashan University of Medical Sciences, Kashan, Iran; ^9^Department of Cell Systems and Anatomy, UT Health. Long School of Medicine, San Antonio, TX, United States; ^10^Laser Research Centre, Faculty of Health Science, University of Johannesburg, Johannesburg, South Africa

**Keywords:** cardiovascular disease, pathophysiology, melatonin, antioxidant, cardiotoxicity

## Abstract

The pineal gland is a neuroendocrine gland which produces melatonin, a neuroendocrine hormone with critical physiological roles in the circadian rhythm and sleep-wake cycle. Melatonin has been shown to possess anti-oxidant activity and neuroprotective properties. Numerous studies have shown that melatonin has significant functions in cardiovascular disease, and may have anti-aging properties. The ability of melatonin to decrease primary hypertension needs to be more extensively evaluated. Melatonin has shown significant benefits in reducing cardiac pathology, and preventing the death of cardiac muscle in response to ischemia-reperfusion in rodent species. Moreover, melatonin may also prevent the hypertrophy of the heart muscle under some circumstances, which in turn would lessen the development of heart failure. Several currently used conventional drugs show cardiotoxicity as an adverse effect. Recent rodent studies have shown that melatonin acts as an anti-oxidant and is effective in suppressing heart damage mediated by pharmacologic drugs. Therefore, melatonin has been shown to have cardioprotective activity in multiple animal and human studies. Herein, we summarize the most established benefits of melatonin in the cardiovascular system with a focus on the molecular mechanisms of action.

## Introduction

Cardiovascular disease (CVDs) accounts for the majority of deaths worldwide (1, 2), and is more predominant in older generation (3). According to WHO report, CVDs accounted for 17.9 million deaths in 2019, representing 32% of all global deaths (3–8). Although the mortality rate is now being reduced, the prevalence of CVDs still remains too high (9). The CDC reports that ~610,000 people die due to CVDs each year in the USA (10).

Cardiovascular disease encompasses a group of disorders involving blood vessels or the heart (11), including coronary artery disease, myocardial infarction, angina, hypertensive heart disease, heart failure, cardiomyopathy, arrhythmia, congenital heart disease, valvulopathy, aortic aneurysm, carditis, rheumatic heart disease, venous thrombosis, thromboembolic disease, and peripheral vascular disease (4, 7, 11, 12). Coronary artery disease accounts for the majority of cases, causing ~375,000 deaths per year in the USA (10, 13).

The beneficial effects of melatonin in treating various human diseases has been broadly investigated (14–20). Melatonin is an indoleamine-derived molecule, which is synthesized at night in the pineal gland of the brain under control by the hypothalamic suprachiasmatic nucleus (21–24). The traditional function of melatonin could be exert as an endogenous synchronizer of circadian and seasonal rhythms, which modulates sleep patterns (22, 23). In addition, melatonin exhibits many other biological functions, such as anti-inflammatory, antioxidant, anti-excitatory, immunomodulatory, metabolic, and vasomotor activities (25, 26). In particular, endogenous melatonin plays a significant role in numerous CVDs and metabolic disorders, which can result in the development of heart failure (15, 17, 27–29).

The effects of melatonin in the cardiovascular system have been investigated in several previous studies (30–34). Melatonin has direct interactions with the nervous system, and indirect interactions with blood vessels and the heart (29, 32, 34). Melatonin exerts its direct functions by a receptor-dependent signaling pathway, and its indirect functions as a free radical scavenger (33, 35). The receptors for melatonin are G-protein coupled receptors, such as membrane receptors type 1 (MT1, Mel1A, MTNR1A) and type 2 (MT2, Mel1B, MTNR1B), as well as the retinoid-related orphan nuclear receptors RZR and RORα (26, 33). Upon binding to these receptors, melatonin can exert modulatory effects in the blood vessels and the heart (33). Various signaling pathways have been shown to mediate the downstream effects of melatonin, such as adenylate cyclase, phospholipase C, protein kinase C (PKC), guanylate cyclase, potassium channels, calcium channels, and phospholipase A2. Some of these mediate the anti-adrenergic effects of melatonin (33, 36, 37). Melatonin receptors play an essential role in reducing the risk of heart failure (37–41) and cardiomyopathy (28, 42–44) after myocardial infarction. In this review the authors aim to point out therapeutic potentioals of melatonin in the treatment of CVDs with an emphesis on the molecular mechanisms of action. Moreover, current clinical trials using melatonin in heart disease are discussed.

## The Biological Functions of Melatonin as a Neurohormone and Antioxidant

**Figure 1:**
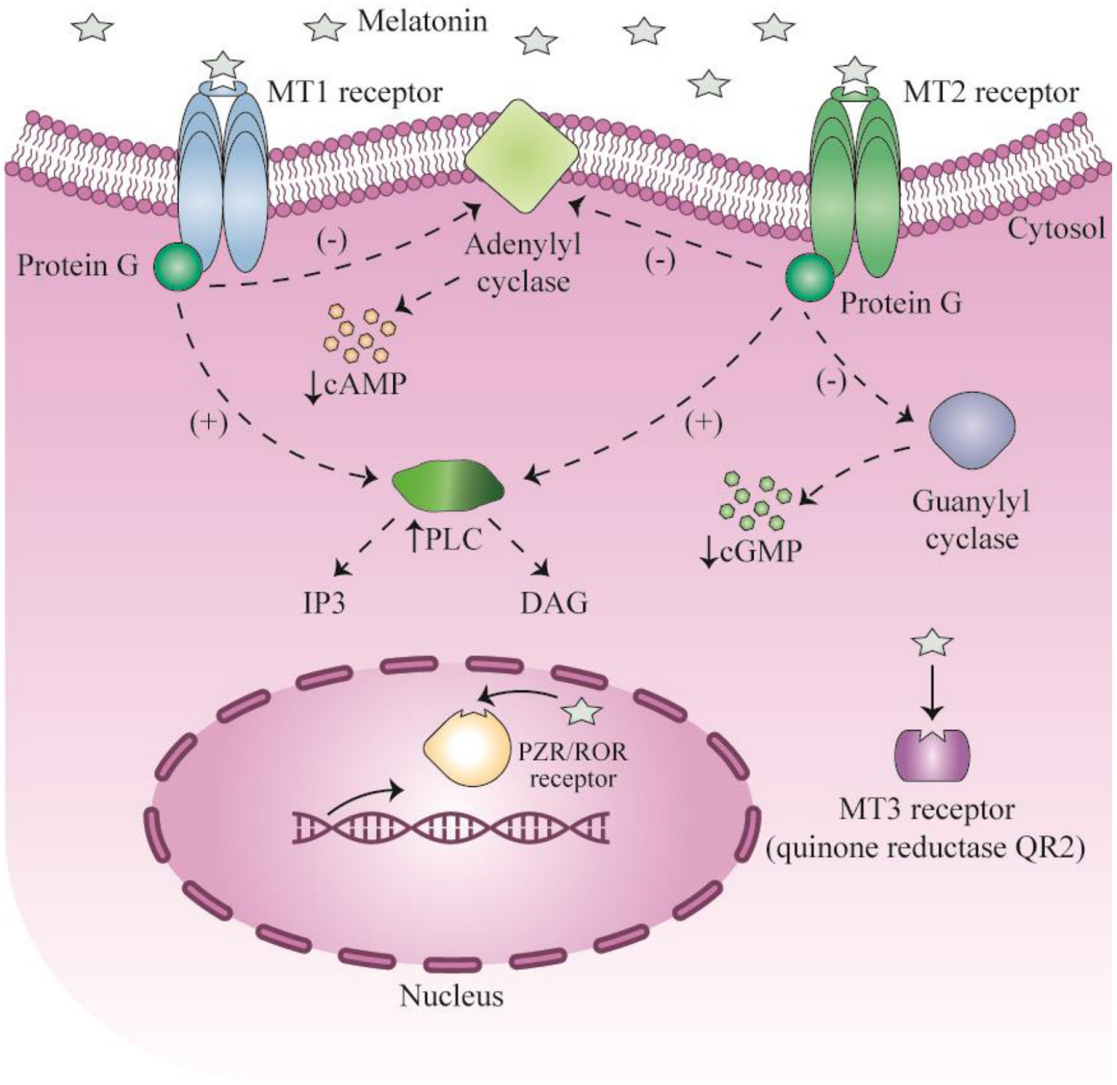
Signaling of melatonin and their receptors. This figure adapted from Millet-Boureima et al. (45).

When melatonin binds to MT1 and MT2 receptors, it exerts its regulatory function on the circadian rhythm, sleep-wake cycle, and body temperature cycles (Figure 1) (46–54). Administration of melatonin to humans results in sleepiness, fatigue and reduced sleep latency (55). Impaired circadian rhythms have been associated with poor health and sleep disorders (56). For instance, pediatric populations with various neuropsychiatric, developmental, or health disorders often exhibit a deficiency of melatonin (57). Following the restoration of melatonin levels, circadian rhythms, as well as developmental, mood, behavioral, and health disorders may be improved. Improved intellectual ability and even control of seizures may be obtained (56, 58). It is known that circadian rhythms are critical for the normal development and function of the nervous system, and their disruption eliminates neurogenesis in laboratory animal models (59–62). Melatonin may also play an important role in embryonic development, with direct effects on the placenta and neuroglial structures. Additionally, melatonin has important functions in several stages of ontogenesis, including establishing diurnal rhythms and synchronization of the biological clock in the fetus (63, 64).

In addition to the well-established role of melatonin in sleep-wake rhythm modulation, melatonin functions as an endogenous chronobiotic and synchronizing agent, which strengthens oscillations and regulates central biological clock timing in the hypothalamic suprachiasmatic nucleus resulting in stable bodily rhythms (65). Moreover, a study by Pevet and Challet (66) showed that melatonin can serve as both an endogenous-time regulator, and can control the master clock output in complex circadian networks. Melatonin transfers temporal signals to various tissues possessing melatonin receptors, which results in the induction and regulation of circadian rhythms in a number of organs. These organs include the adenohypophysis, and peripheral tissues such as the liver, pancreas, lungs, heart, kidneys, adipose tissue, gastrointestinal tract, as well as the fetal adrenal gland. Circadian rhythms and the circadian clock network enable biological processes to be temporally organized in response to episodic environmental changes, thus providing environmental adaptability (67).

Melatonin exerts its anti-oxidant effects *via* a cell surface receptor-independent pathway, since the MT3 receptor is a QR2 (quinone oxidoreductase 2) cytosolic enzyme. Melatonin reduces the generation of new free radicals and reactive oxygen species (ROS). The ability of melatonin to scavenge ROS was present in mammalian species from the evolutionary period (68). Melatonin acts as a natural antioxidant and scavenger, and can reduce both reactive nitrogen species and ROS (69). Melatonin binds directly to the cytosolic QR2 catalytic site, and regulates the function of QR2 in order to detoxify or reduce production of ROS (70, 71). The detoxification mediated by melatonin maintains redox homeostasis in cells, and protects cells against damage and oxidative stress (72). The QR2 MT3 receptor is involved in protection against neurodegeneration in brain cells, and reduces ulceration and carcinogenesis in the gastrointestinal tract.

The pineal gland is also involved in the regulation of the immune system responses (73), as shown by the reduced cell-mediated immunity and humoral responses following pharmacological inhibition (administration of propranolol) or functional inhibition (constant light condition) of melatonin synthesis in mouse models. The immune system and the pineal gland display bidirectional interactions, because cytokines, interleukins, and interferon-γ can alter the production and release of melatonin (74). The transcription factor NF-κB plays a crucial role in inflammatory responses. NF-κB activity is typically blocked by binding of a specific protein IκB (inhibitor of NF-κB). In inflammation, the release of pro-inflammatory mediators, ROS generation, and TLR (toll-like receptor) activation stimulates a kinase enzyme (IκB kinase), which phosphorylates IκB and promotes its dissociation from NF-κB, allowing NF-κB to translocate to the nucleus. In the nucleus, NF-κB regulates the transcription of many pro-inflammatory genes. Melatonin suppresses the activation as well as the translocation of NF-κB in various cells, including T cells, neuronal cells and macrophages (75–77). There are two RAR Related Orphan Receptors (RORγ and RORα), which can modulate the inflammatory response. RORγ stimulates Th17 cell lineage differentiation, and modulates the expression of numerous pro-inflammatory mediators (IFN-γ, IL-17F, IL-17, IL-2, and TNF-α) (78, 79). Melatonin binds to ROR/RZR nuclear receptors, and affects gene transcription and thus inflammatory responses (80).

Melatonin has also been shown to have anti-cancer activity in several previous studies, and has the potential to protect against tumorigenesis. Melatonin has shown anti-proliferative effects, anti-oxidant properties, and can activate the anti-tumor immune response. However, there are some studies showing that melatonin can promote tumor formation and growth, particularly when melatonin is given in the morning. These observations suggest that the anti-cancer activity of melatonin may be dependent on the stage of the circadian cycle (81). In metastatic non-small cell lung carcinoma, a controlled clinical trial found that simultaneous administration of cisplatin and etoposide combined with melatonin could improve treatment outcomes in terms of both quality of life and survival rate (82).

**Figure 2:**
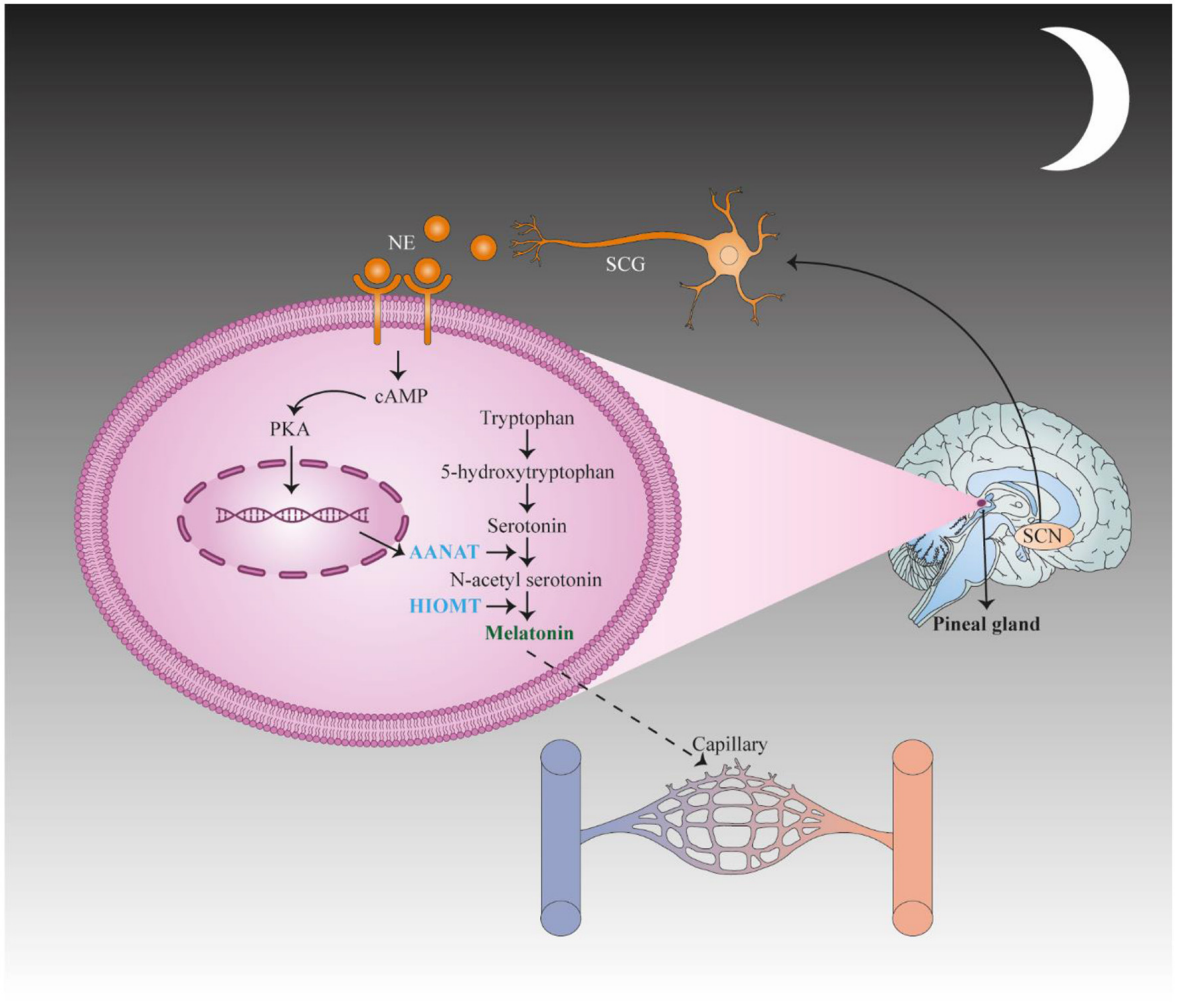
First, the suprachiasmatic nucleus (SCN) translates stimulation to the spinal cord and the superior cervical ganglia (SCG) of the sympathetic nervous system and subsequently activates adrenergic fibers to secrete norepinephrine (NE), which binds to adrenergic receptors in pinealocytes. These changes result in the up regulation of N-acetyltransferase (AANAT), the key enzyme in melatonin synthesis, *via* activating CAMP signaling. This change causes an increase in the concentration of N-acetyl serotonin, which is converted to melatonin by hydroxyindole-O-methyltransferase (HIOMT). This figure adapted from Song et al. (83).

Reduced overall production of melatonin and dysregulated nocturnal synthesis of melatonin have been associated with several disorders of the CNS (central nervous system), including schizophrenia, obsessive-compulsive disorder, and stroke (Figure 2) (84). The human brain accounts for only 2% of total body weight, but consumes 20% of the overall intake of oxygen and glucose in the body. Cells in brain tissue produce more ROS in comparison with other tissues. There is a high concentration of ascorbic acid and polyunsaturated fatty acids in brain tissue, which are vulnerable to free radical-mediated injury, when enzymatic antioxidants are inadequate. Melatonin has a neuroprotective effect in several diseases, including amyotrophic lateral sclerosis, epilepsy, Parkinson's disease, Alzheimer's disease, traumatic brain injury, and brain ischemia (85, 86). In the majority of these disorders, there is a progressive loss of neurons, accompanied by mitochondrial dysfunction, glutamate excitotoxicty, and free radical damage (86).

Melatonin has many beneficial effects on cardiovascular system. In this context, melatonin can regulate heart rate (87), and reduce nocturnal blood pressure in patients with hypertension (88, 89). Moreover, melatonin may serve as a potent protective agent in the cardiovascular system, and diminish the risk of developing reperfusion injury after myocardial infarction (90). The benefits of melatonin are related to its ability to scavenge free radicals, reduce oxidative stress, modulate metabolic activity, regulate production of cytokines, and prevent against apoptosis. These findings have been confirmed by various preclinical and animal studies (91, 92). Nevertheless, a larger number of clinical studies are required to confirm these benefits in humans. Beta-blockers are often administered to patients with hypertensive disorders, and these drugs can block endogenous secretion of melatonin. Consequently, hypertensive patients may experience insomnia as an adverse effect of beta-blockers. Administration of exogenous melatonin supplements can improve the quality and amount of sleep in these patients (93).

Melatonin can affect multiple cardiovascular functions, such as cardiac output, blood pressure, heart rate, and seasonal rhythms. The functions of melatonin are related to the activity of the melatonergic system and the suprachiasmatic nucleus (94). After resection of the pineal gland, the essential source of melatonin circulating in plasma, the blood pressure in rats was elevated, while administration of melatonin to rats with hypertension can reduce arterial pressure, baroreflex response, and heart rate. The mechanism for this effect involves a decrease in CAMP and an increase in hydrolysis of phosphatidylinositol-4,5-bisphosphate (95). It has also been speculated that melatonin can activate endothelial cells *via* binding to MT2 receptors, leading to the synthesis of nitric oxide which promotes the generation of soluble guanylate cyclase in smooth muscle cells. This leads to an increase in cGMP production and hence to vasodilation. ROS and reactive nitrogen species are known to contribute to the pathogenesis of cardiac ischemic reperfusion injury. Melatonin exerts its ROS scavenging function in mitochondria, with beneficial effects in ischemic heart disease and prevents ischemia reperfusion-mediated myocardial damage. Moreover, melatonin shows therapeutic efficacy in vasculopathy caused by nicotine. Administration of nicotine has been associated with significant endothelial injury and aortic vasoconstriction, which can be counteracted by melatonin (96, 97). The reduced levels of superoxide dismutase and nitric oxide synthase caused by chronic nicotine administration may also be improved by melatonin.

## Melatonin Protective Effects Against Drug-Induced Cardiotoxicity

Doxorubicin (Dox) is a commonly used anticancer chemotherapeutic drug in the class of anthracyclines, which has been extensively used in the management of both hematologic and solid tumors (98). However, the therapeutic application of Dox has been limited, because it causes cardiac hypertrophy and heart failure. A study showed that administration of trastuzumab plus anthracyclines in patients led to increased left ventricular afterload and preload, whereas it resulted in reduced strain, heart rate and ejection fraction. In addition, recovery from these effects was not observed even after a 2-year-follow up (99, 100). Dox produces cardiac toxicity *via* multiple pathways, including a large increase in ROS proudction (101). Myocytes have abundant mitochondria which are needed for production of sufficient ATP for heart contraction, and maintaining cell viability in the myocardium (102, 103). Dox accumulates in the mitochondria, where it damages the mitochondrial membrane, reducing the MMP (mitochondrial membrane potential) and producing ROS, leading to the death of cardiomyocytes (104). In addition, studies have found that Dox can trigger apoptosis by decreasing the anti-apoptotic protein Bcl2, and inceasing pro-apoptotic Bax (105–107).

Yes-associated protein (YAP) is a down-stream component of the Hippo signaling pathway, which affects several cardiac physiological and pathological processes. These include the development of cardiac muscle, formation of new blood vessels, cellular apoptosis, autophagy, hypertrophy, and metabolic homeostasis (108). Suppression of YAP leads to aggravated heart failure after myocardial infarction, and apoptosis and fibrosis in cardiac muscle cells (109). In previous studies, YAP was found to decrease myocardial injury after MI, and increase the survival of cardiomyocytes by increasing the expression of YAP-target genes, which ultimately improves heart performance. Activation of genes involved in the cell cycle are involved in the YAP response (110). Both post-MI heart failure and non-ischemic heart failure are related to the Hippo signaling pathway. Systolic heart failure following MI can be inhibited by suppression of the Hippo pathway (111). Furthermore, YAP can regulate the antioxidant activity within heart cells. Inhibition of YAP expression leads to suppression of the activity of FoxO1 transcription factor and lower expression of anti-oxidant genes, which in turn worsens myocardial ischemia-perfusion injury (112).

Li et al. carried out a study to explore the potential anti-oxidant effects of melatonin, and whether it could protect against apoptosis mediated by Dox in cardiac muscle cells (113). Their study showed that treatment with Dox increased apoptosis and decreased viability of H9c2 cells. They found increased TUNEL positive cells, elevated expression of Bax, and reduced expression of Bcl2. This effect was linked to decreases in MMP, and increases in ROS. Treatment with Dox for 5 weeks was associated with significant LV dysfunction detected by echocardiography *in vivo*. Although Dox-mediated apoptosis was higher by TUNEL staining, concomitant administration of melatonin and Dox reduced ROS generation in cardiomyocytes, and inhibited apoptosis by increasing the MMP. Moreover, melatonin-Dox combined therapy decreased Dox-induced cardiac damage *in vivo*. *In vivo* immunohistochemistry staining, *in vitro* immunofluorescence, and Western blotting showed that treatment with Dox significantly suppressed expression of YAP, while YAP levels were unchanged following concomitant administration of melatonin plus Dox. The protective effects of melatonin against toxicity in cardiac muscle cells were counteracted by YAP suppression mediated by siRNA, which led to increased ROS and apoptosis. Taken together, melatonin therapy decreased the cardiotoxicity mediated by Dox through maintaining YAP levels, leading to lower apoptosis and oxidative stress (113).

AGO (agomelatine) is a small molecule anti-depressant drug, but also acts as an M1 and M2 melatonergic receptor agonist, and 5-HT2C serotonergic receptor antagonist (114). AGO shows marked affinity for M2 and M1 receptors, and has similar antioxidant properties to melatonin (115). AGO protects against ischemia-reperfusion injury by improving anti-oxidant capacity (116).

Aygun and Gul performed a study to evaluate the cardioprotective properties of AGO, melatonin, and AGO + melatonin combined against Dox-induced cardiotoxicity using electrocardiographic, biochemical, and scintigraphic methods (117). In this study, 49 male Wistar rats were randomly allocated to seven different groups, namely control, AGO, melatonin, Dox, Dox + AGO, Dox+melatonin, and Dox + AGO + melatonin. AGO and melatonin were administered intraperitoneally to rats at a dose of 40 mg/kg/day for 7 days; doxorubicin was administered intraperitoneally at 18 mg/kg/day on days 5–7 to induce cardiotoxicity. They carried out 99mTc PYP (technetium-99m pyrophosphate) scintigraphy and ECG (electrocardiography) on the 8th day of the study, in addition to biochemical measures, like BUN, cardiac troponin T (cTnT), and creatine kinase (CK) in the rats. To define acute cardiotoxicity induced by Dox, the following criteria were used: ECG disturbance (reduced duration of QRS and p, ST-elevation, and increased QT and RR intervals), elevated serum CK, BUN and cTnT1, and enhanced uptake of 99mTc PYP in the heart. Pretreatment with AGO, melatonin, or AGO+melatonin could efficiently restore Dox-induced ECG abnormalities to near normal (*p* < 0.001). Furthermore, 99mTc PYP uptake and serum biochemical markers showed that pretreatment with AGO, melatonin, or AGO + melatonin showed equal protective benefits against Dox-induced cardiotoxicity (*p* < 0.001). Their study showed that 99mTc PYP could be an appropriate non-invasive method to monitor Dox-mediated cardiotoxicity (117).

AMPK (adenosine monophosphate activated protein kinase) is a serine/threonine protein kinase (118), which plays a crucial role in regulating endogenous defense mechanisms in cardiomyocytes (119–121). PGC1α controls mitochondrial energy homeostasis by modulating expression of genes, such as UCP2, TFAM, and NRF1 (122, 123). Previous studies have shown that treatment with Dox suppresses the PGC1α and AMPK signaling pathways, leading to exacerbation of myocardial injury (120–122). On the other hand, melatonin activates PGC1α and AMPK signaling pathways, and results in protection of myocardial mitochondrial function (38, 124, 125).

In a study by Liu et al. C57BL/6 mice and H9c2 cells were used to assess the effects of melatonin against acute cardiotoxicity caused by Dox. They studied mitochondrial function, cell viability, apoptosis, oxidative stress, and the AMPK/PGC1α pathway activity (99). Dox caused acute cardiotoxicity in both C57BL/6 mice and H9c2 cells, which was ameliorated by melatonin both *in vivo* and *in vitro*. Melatonin suppressed Dox-mediated mitochondrial dysfunction, apoptosis, cellular morphological abnormalities, and oxidative stress, through activating the AMPK/PGC1α axis, and its target genes (UCP2, NRF1, and TFAM). *In vitro*, either PGC1α siRNA or AMPK siRNA were able to block these effects. *In vivo*, the AMPK inhibitor compound C also abrogated the benefits of melatonin. They concluded that melatonin improved Dox-induced acute cardiotoxicity by activating the AMPK/PGC1α pathway (99).

Reiter et al. (126) reviewed the high concentration of melatonin in the mitochondria, and its antioxidant effects. Previous studies have suggested that mitochondria originally entered eukaryotic cells by the process of endosymbiosis. Additionally, mitochondria have been found to have the potential to effectively synthesize melatonin (127). Melatonin can feasibly diffuse across biological membranes due to its amphiphilic nature, however the high intra-mitochondrial concentration was found to be related to localized mitochondrial membrane transporters, peptide transporters 1 and 2 (PEPT1/2) (128). Melatonin was suggested to be superior to commonly used mitochondrial antioxidants, since its metabolites including N [1]-acetyl-5-methoxykynuramine (AMK) and N1-acetyl-N2-formyl-5-methoxykynuramine (AFMK) also show antioxidant properties, in addition to melatonin itself. Therefore, melatonin may exert its antioxidant effects in a cascade manner (129, 130). Melatonin serves to promote the antioxidant defense systems of the organism, as both pharmacological and physiological doses of melatonin can promote the expression of genes associated with antioxidant activity (CAT, GPx, SOD, GRd) (131–133). Moreover, melatonin (in contrast with the vast majority of small molecule antioxidants) is not able to carry out redox cycling (134). The majority of antioxidants can also act as pro-oxidants by producing relatively stable free radicals, which can then generate additional free radicals. Considering the electron-rich structure of melatonin, it can covalently bind to free radicals producing stable water-soluble molecules (134). As a result, melatonin is a suicidal/terminal antioxidant (as distinct from other antioxidants) (134).

Furthermore, several studies have assessed the effects of melatonin combined with various chemotherapeutic drugs in patients with advanced stage cancer who have an unfavorable clinical prognosis. According to these studies, melatonin could significantly enhance the efficiency of chemotherapy, and reduce Dox-related cardiotoxicity (135–137). In this context, melatonin has both cardioprotective and anticancer properties. The cardioprotective potential of melatonin against Dox-mediated cardiotoxicity is most likely due to indirect antioxidant activity combined with direct free radical scavenging properties. Comprehensive studies on the effects of melatonin on mitochondrial bioenergetics, mitochondrial fusion and fission, cell death and mitophagy, and mitochondrial sirtuin, would improve our understanding of the protective mechanisms against Dox-induced cardiotoxicity.

Govender et al. demonstrated the effects of melatonin on cell death, mitochondrial fission and fusion, cardiac function, sirtuin and PGC1-α expression, in a rat model of acute Dox-mediated cardiotoxicity *in vivo*. Moreover, they investigated ATP synthesis and mitochondrial structure in acute Dox-induced cardiotoxicity *in vitro*. According to their results, administration of melatonin prior to Dox treatment can preserve mitochondrial function in DOX-mediated cardiotoxicity, and improve survival of cardiomyocytes (138). *In vitro* H9c2 rat cardiomyoblasts received melatonin as pre-treatment (10 μM, 24 h), which was followed by administration of Dox (3 μM, 24 h). Mitochondrial structure and ATP levels in cells were evaluated. Dox caused cell death and fission of mitochondria, both of which were decreased following administration of melatonin. The *in vivo* study employed Sprague Dawley rats bearing breast cancer tumors (LA7) which received a Dox injection with or without melatonin in their drinking water for 14 days. Rats receiving melatonin in combination with Dox showed higher cardiac output in comparison to rats receiving Dox alone. The mean tumor volume on day 8 was remarkably lower in rats receiving melatonin plus Dox, compared to rats treated with Dox alone. The combined melatonin and Dox treatment was associated with higher intracellular ATP levels, SIRT1, and PGC1-α expression levels, compared to Dox alone. They concluded that melatonin provides a dual anticancer and cardioprotective benefit by increasing mitochondrial and cardiac functions (138).

The anticancer effects of melatonin have been shown in numerous tumor types, such as ER+ breast cancer (139–142). SOD (superoxide dismutase) is an enzyme which produces hydrogen peroxide and oxygen molecules from superoxide radicals, and thus plays a major role in protecting against cellular injury mediated by ROS. GPx (glutathione peroxidase) is another antioxidant enzyme extensively found *in vivo*. This enzyme serves as an antioxidant, preventing the accumulation of lipid peroxides (LPO) in cellular membrane. LPO are generated from polyunsaturated fatty acids present in cell membranes. Cell membrane oxidative damage has been associated with a wide range of diseases.

Floyd et al. reported that LPO, GPx, and SOD to be involved in Dox induced cardiotoxicity (143). They observed a negative correlation between GPx and SOD and the damage induced by Dox, meanwhile LPO showed a positive correlation. Melatonin has demonstrated a significant role in protection against lipid peroxidation and oxidative membrane damage. This mechanism may explain why melatonin protects against Dox associated myocardial injury (144–147).

In a study by Zhang et al. the cardioprotective properties of melatonin and its role in enhancing the anticancer effects of Dox were investigated. They used a model of rat ER^+^ breast tumor to study the effects of melatonin (148). After induction of the breast tumor, they randomly distributed the rats between five groups: no treatment control, solvent [dehydrated alcohol: physiological saline (1:9)], melatonin alone, Dox alone, melatonin + Dox (M + D). They measured LPO, GPx, and SOD levels in the myocardium, myocardial tissue was evaluated by electron and light microscopy, and they followed the survival rates of the different groups for a 1-month period. Breast tumor was identified in 116 rats. In comparison to the control group, the group receiving Dox showed high lipid peroxides, whereas GPx and SOD activity were considerably lower. M+D group had higher GPx and SOD activity (*P* < 0.05), whereas lipid peroxide was lower than Dox alone (*P* < 0.05). Moreover, the rats group exposed to M + D had less significant myocardial damage compared to Dox alone, and 1-month life expectancy was higher in the group receiving M + D in comparison with Dox alone. Consequently, melatonin can decrease oxidative damage mediated by Dox in myocardial tissue and play a cardioprotective role (148).

Table 1 lists some studies on the effects of melatonin on drug-induced cardiotoxicity and heart damage.

**Table 1:** Effects of melatonin on drug-induced cardiotoxicity and protection against heart damage.

**Drug or agent**	**Melatonin dose**	**Treatment duration**	**Biochemical measures**	**Effects**	**Model**	**References**
Doxorubicin	4 mg/kg	2 days	Malondialdehyde (MDA)	Protected the heart from dox-induced damage	*In vivo* (rat)	(149)
Doxorubicin	10 mg/kg	7 days	MDA, glutathione (GSH)	Prevented lipid peroxidation and myocardial lesions	*In vivo* (rat)	(150)
	10 mg/kg	120 h, 3 weeks	MDA, 4-hydroxyalkenals	Significantly reduced cardiac muscle lesions	*In vivo* (rat)	(151)
Daunorubicin	10 mg/kg	120 h, 3 weeks	MDA, 4-hydroxyalkenals	Significantly reduced cardiac muscle lesions	*In vivo* (rat)	(151)
Doxorubicin	50 μg/kg	10 days	MDA, GSH, 4-hydroxyalkenals	Reduced oxidative damage	*In vivo* (rat)	(152)
Doxorubicin	10 mg/kg	5 weeks	Yes-associated protein (YAP)	Attenuated Dox-induced cardiotoxicity, decreased oxidative stress, & apoptosis	*In vivo* (mice)	(153)
Doxorubicin	5 mg/kg	10 days	Troponin I, leptin, triglycerides, cholesterol, LDL-cholesterol, T3, T4, and IL-1a	Reduced oxidative stress, activated antioxidant enzymes in cardiac cells	*In vivo* (rat)	(154)
Doxorubicin	10 mg/kg	5 days	–	Protected against Dox-induced cardiotoxicity without interfering with its antitumor effect	*In vivo* (mice)	(155)
Doxorubicin	40 mg/kg/day	7 days	BUN, CK, cTnT	Reversed cardiac damage caused by Dox	*In vivo* (rat)	(117)
Doxorubicin	20 mg/kg	4 weeks	Glutathione peroxidase (GPx), SOD, catalase (CAT), GSH, MDA, 4-HDA	Blocked cardiac injury caused by Dox	*In vivo* (rat)	(156)
Doxorubicin	20 mg/kg	7 days	AMPK/PGC1α	Attenuated DOX-induced cardiac dysfunction and pathological changes	*In vivo* (mice)	(99)
Doxorubicin	10 mg/kg	7 days	–	Protected against Dox-induced cardiotoxicity	*In vivo* (rat)	(157)
Doxorubicin	10 mg/kg	15 days	LPO, SOD, GPx	Reduced Dox-induced cardiac oxidative damage	*In vivo* (rat)	(148)
Doxorubicin	6 mg/kg	14 days	PGC1-α, Sirtuin	Suppressed oncogenesis and cardiac damage through enhancing mitochondrial function	*In vivo* (rat)	(138)
Epirubicin	200 μg/kg	10 days	MDA, nitric oxide (NO), GSH, fibronectin, laminin	Suppressed epirubicin-induced nitrosative stress, reduced degeneration in heart tissue	*In vivo* (rat)	(158)
Doxorubicin	10 mg/kg	6 days	MDA, lactate dehydrogenase (LDH), serum creatine kinase	Protected against Dox-induced cardiotoxicity and enhanced its antitumor activity	*In vivo* (rat)	(137)
Doxorubicin	84 mg/kg	3 weeks	Thiobarbituric acid reactive substances (TBARS)	Significantly decreased heart to body weight ratio, arterial pressure, left ventricular fractional shortening, reversed Dox-induced cardiomyopathy	*In vivo* (rat)	(159)
Doxorubicin + trastuzumab	10 mg/kg	5 days	MDA, SOD, GPx, serum creatine phosphokinase (CK-MB)	Significantly reversed oxidative stress markers	*In vivo* (rat)	(160)
Doxorubicin	1, 5 mg/kg	5 days	Non-protein sulfhydryls (NP-SH), nitrate/nitrite (NO), plasma aminotransferases, LDH, CK-MB	Inhibited Dox-induced lipid peroxidation in heart, liver, and kidney	*In vivo* (Mice)	(161)
Cyclosporine A	1 mg/kg/d	21 days	Thiobarbituric acid reactive substances (TBARS), GSH, CAT, SOD	Increased antioxidant enzymes, normalized cardiac morphology	*In vivo* (rat)	(162)
Doxorubicin	10 mg/kg/d	7 days	CK, CK-MB, AST, LDH, SOD, GPx, MDA	Inhibited Dox-induced cardiac damage	*In vivo* (rat)	(163)
Epinephrine	50 μM	10, 15, 20 min	–	Cardioprotective effects	*In vivo* (rat)	(164)
Doxorubicin	5 mg/kg/d	30 days	GSH, SOD	Protected against Dox-induced cardiotoxicity, enhanced Dox antitumor activity	*In vivo* (mice)	(165)
Doxorubicin	6 mg/kg	15 days	TBARS, conjugated dienes (CD)	Protected against Dox toxicity	*In vivo* (mice)	(166)
Doxorubicin	1 mM	1 h	LDH	Protected against Dox induced mitochondrial damage	*In vitro* (primary myocytes)	(167)

## Melatonin and Stem Cell Therapy for Heart Regeneration: Synergistic Effects on Cardiac Progenitor Cells

Myocardial infarction (MI) is the most common cause of morbidity and mortality globally. Despite the success of surgical intervention and pharmacological therapy, which have reduced MI-related mortality, the heart does not possess the ability to naturally regenerate itself, and hence cardiac function is often impaired in the long term after MI (168). Stem cells possess the potential for multi-lineage differentiation, and combined with paracrine signaling, their transplantation offers a potential treatment for the regeneration and repair of injured cardiac and vascular tissue following MI (169). Nonetheless, transplanted stem cells are prone to death by necrosis and/or apoptosis within the ischemic cardiac muscle, and the presence of inflammatory mediators and oxidative stress in the infarcted region can significantly limit the efficiency of stem cell transplantation (170, 171). Several techniques have been proposed for improving stem cell viability after transplantation into infarcted heart tissue (172). For example, the survival rate of stem cells was increased by transferring genes encoding anti-apoptosic proteins, like Bcl-2 (173) or survivin (174). Several small molecule chemical compounds, like melatonin have also been tested for this purpose (175). Moreover, other tissue engineering techniques such as cellular aggregates or cellular sheets, have been tested to improve the survival rate in transplanted stem cells (176, 177). Among the approaches listed above, pre-treatment with a natural supplement (melatonin) may be more practical and economical.

According to previous studies, melatonin pre-treated stem cells show a higher resistance to oxidative stress damage, and several mechanisms have been proposed to explain this observation, including direct ROS detoxification, and indirect stimulation of antioxidant defense enzymes (126, 178–180). However, melatonin does not apprear to exert a prolonged protective effect on transplanted stem cells to ensure their long-term engraftment (181).

Nanoscale drug delivery carriers can regulate the release of drugs from polymeric nanoparticles, to enhance bioavailability and decrease dosage to avoid adverse effects (182). This approach could be used for drug pretreatment before strem cell transplantation. Nonetheless, whether nano drug delivery carriers for melatonin are more effective compared to free melatonin, had not been tested.

Ma et al. (183) described the preparation of melatonin nanoparticles (Mel-NPs) by encapsulating melatonin inside the biodegradable, non-antigenic, and non-toxic polymer, PLGA-mPEG. The protective effects and the underlying mechanisms of the melatonin nanoparticles were investigated. A hypoxia/reperfusion (H/R) model was utilized to reproduce the oxidative stress microenvironment following MI. They evaluated the association between p53-cyclophilin D (CypD) complex, which controls mPTP (mitochondrial permeability transition pore) opening and melatonin. In their study, the protective effects of melatonin nanoparticles (Mel-NPs) on adipose derived stem cells (ADSC) were evaluated and compared to those of melatonin alone *in vitro* and *in vivo*. *In vitro*, Mel-NPs inhibited the p53-cyclophilin D complex, suppressed mPTP opening, and alleviated H/R damage in ADSCs. In addition, Mel-NPs resulted in longer survival rates of ADSCs in infarcted myocardial tissue of rats, compared to free melatonin, and the therapeutic benefits were more pronounced. Taken together, the combined approach of stem cell transplantation and Mel-NPs for treament of MI, may be a novel and efficient strategy (183).

The normal protein PrPC (cellular prion protein) is an ubiquitous glycoprotein anchored to the cell membrane *via* glycosylphosphatidylinositol, which is conserved across species (184). Despite the fact that the abnormal prion protein (PrP) plays a role in pathogenesis of neurodegenerative disorders and prion diseases (185), accumulating evidence suggests that the normal PrPC plays a major role in the proliferation and self-renewal of stem cells (186–188), and could enhance their protective role against neurodegenerative disorders (189). Several studies have shown that PrPC has a critical function in the differentiation of progenitor and/or stem cells (190, 191), neurogenesis (188, 192), and formation of blood vessels (193). Nevertheless, the underlying mechanism by which PrPC protects transplanted stem cells in various pathophysiological disorders, remains poorly understood.

Lee et al. investigated the beneficial effects of melatonin in improving the biological activity of mesenchymal stem cells in the ischemic myocardium. Their study showed that melatonin could increase the expression of PrPC, which in turn regulated resistance to oxidative stress, proliferation, and the immunomodulatory properties of mesenchymal stem cells. Subsequently, the potential capacity of melatonin activated mesenchymal stem cells to promote neovascularization was evaluated in a mouse model of hind-limb ischemia (194). Administration of melatonin promoted the proliferation of mesenchymal stem cells and self-regeneration by increasing the expression of PrPC. Furthermore, melatonin decreased apoptosis in mesenchymal stem cells during oxidative stress by different mechanisms, such as regulating apoptotic proteins, caspase-3, PARP-1, BCL-2, and BAX in a PrP^C^-dependent manner. Additionally, melatonin could modulate the immunomodulatory properties of mesenchymal stem cells through the PrP^C^-IDO (Indoleamine 2,3-dioxygenase) axis. Furthermore, melatonin stimulated stem cells improved limb salvage, blood flow perfusion, and angiogenesis while lowering macrophage infiltration in the model of hind-limb ischemia. The therapeutic effects of melatonin were suppressed by blocking the expression of PrP^C^. According to their study, melatonin could enhance the performance of mesenchymal stem cells and stimulate angiogenesis in ischemic tissues *via* increasing the expression of PrP^C^. Melatonin-mediated PrPC targeting may provide a novel treatment approach in mesenchymal stem cell therapy (194).

As mentioned above, melatonin has been shown to stimulate antioxidant enzymes, like SOD and catalase, which could increase mesenchymal stem cell resistance to apoptosis induced by hydrogen peroxide (195, 196). Regulating the ischemic environment through inhibiting excessive inflammation and oxidative damage could improve the efficacy of mesenchymal stem cell transplantation in ischemic tissues (197, 198).

Han et al. performed a study to assess whether the cardioprotective effect of AD-MSCs (adipose-derived mesenchymal stem cells) could be promoted by melatonin (199). The mechanism of action of melatonin on SIRT1 signaling was evaluated in a cell model of hypoxia/serum deprivation (H/SD) *in vitro*. SIRT1 or sirtuin 1 (silent mating type information regulation 2 homolog 1) is a deactylating enzyme in the nucleus that activates transcription factors. *In vivo*, melatonin increased transplanted AD-MSC survival as well as promoting cardiac function following MI. They demonstrated that melatonin could enhance the survival rate of AD-MSCs in the ischemic myocardium and synergistically improve cardiac function in combination with AD-MSCs. Melatonin resulted in less oxidative stress, apoptosis and inflammation in the ischemic tissue *in vivo*. Mechanistically melatonin may promote SIRT1 signaling, resulting in an increase in Bcl2 and inhibition of Bax, Ac-p53, Ac-NF-κB and Ac-FoxO1. Therefore, melatonin may be a promising treatment strategy to improve MSC therapy in ischemic cardiac disease, by regulating SIRT1 signaling (199).

Table 2 lists some studies on the synergistic effects of melatonin and progenitor cells for cardiac regeneration.

**Table 2:** Studies on the synergistic effects of melatonin and progenitor cells for cardiac regeneration.

**Type of stem cell**	**Stem cell origin**	**Melatonin dose**	**Target**	**Effect**	**Model**	**References**
Mouse embryonic stem cells	–	100 μM, 100 nM	Hypoxia inducible factor (HIF)	Promoted cardiac differentiation and maturation of mESCs	*In vitro*	(200)
Mesenchymal stem cells	Mouse adipose tissue	5 μM	Catalase, Cu/Zn SOD, IGF-1, basic fibroblast growth factor, hepatocyte growth factor (HGF), EGF	Inhibited H_2_O_2_ induced apoptosis in MSCs	*In vitro*	(196)
				Improved viability of engrafted MSCs in cardiac tissue	*In vivo*	
Mesenchymal stem cells	Mouse adipose tissue	20 mg/kg/d	SIRT1	Enhanced viability of cardiac transplanted AD-MSCs, synergistically increased cardioprotective effects	*In vivo*	(199)
Mesenchymal stem cells	Bone marrow	5 μM	–	Increased proliferation of MSCs, improved LVEF & LV wall thickness	*In vivo*	(201)
Mesenchymal stem cells	Adipose tissue	20 mg/kg/d	Cellular prion protein (PrPC)	Enhanced MSC proliferation & self-renewal *via* upregulation of PrPC	*In vivo*	(194)
Mesenchymal stem cells	Adipose tissue	0.5 mM		Melatonin-nanoparticles improved ADSC survival rates, & efficiency of stem cell transplantation	*In vivo*	(202)
Mesenchymal stem cells	Bone marrow	10–200 nM	Phospho-P38MAPK & phospho-ERK1/2	Improved survival of MSCs in hypoxia and serum deprivation condition	*In vitro*	(203)

## Melatonin for Ischemia-Reperfusion Injury and Preventing Myocardial Damage

Two melatonin receptor subtypes (MT1 and MT2) are present in mammalian cells. Both of these receptors are coupled to Gi/o-type proteins, while MT1 is also coupled to Gq-type proteins (80, 204). Melatonin affects a wide range of physiological processes in mammals, by activating membrane receptors (80, 205). Melatonin membrane receptors in the myocardium have been shown to regulate numerous survival signaling pathways, such as SIRT1 and Hes1 (33, 206, 207). Nevertheless, the exact mechanism by which melatonin exerts its effects in the myocardium is still poorly understood. Melatonin receptors can modulate various signaling pathways within the cells, including cAMP, PKA (protein kinase A), PKG (protein kinase G), cGMP, and PLC (phospholipase C) signaling pathways (80, 204, 205). Amongst the pathways listed above, PKG and cGMP signaling have been shown to act as major mediators of cardiac protection (208). cGMP-PKG may be involved in cardioprotective pathways in MI or reperfusion injury, particularly in patients with diabetes (209–211). Up to now, how exactly melatonin regulates cGMP-PKG remains to be understood. Melatonin may decrease or increase the intracellular levels of cGMP, depending on the pathological or physiological conditions, and on the cell type (212–215). Nrf-2 (nuclear factor-erythroid factor 2-related factor 2) is a transcription factor that modulates several antioxidant genes and protective enzymes, which is ubiquitously expressed in the cardiovascular system (216). In previous studies, Nrf-2 and its target HO-1 (heme oxygenase-1) showed protective effects against MI and reperfusion injury in patients with diabetes (216, 217). In several different organs, an association between Nrf-2/HO-1 and cGMP-PKG activity has been demonstrated (218). Additionally, the cGMP-PKG axis could regulate the MAPK (mitogen-activated protein kinase) cascade in ischemic myocardium. Alterations in JNK, ERK, and p38 kinase, could modulate apoptosis in cardiac muscle cells (219–221). Nevertheless, it is not clear if these signaling pathways play a role in melatonin-related cardioprotection or how they interact with melatonin membrane receptors.

Yu et al. designed a study to evaluate how cGMP-PKG, the Nrf-2-HO-1 axis, and the MAPK cascade were involved in the cardioprotective effects of melatonin (222). They used an *in vivo* model of Sprague-Dawley rats with diabetes induced by streptozotocin, while *in vitro* studies used H9c2 cardiomyoblasts incubated in high-glucose medium. Melatonin increased intracellular levels of cGMP, expression of PKGIα, the p-VASP/VASP ratio, as well as regulating the MAPK and Nrf-2-HO-1 signaling pathways in the myocardium. These effects were abrogated by KT5823, which is a selective PKG inhibitor, or by PKGIα siRNA, with the exception of intracellular cGMP levels, which remained unchanged. In addition, their study showed that 4P-PDOT (selective antagonist of MT2 receptor) or luzindole (non-selective antagonist of melatonin receptors) suppressed the protective property of melatonin, and prevented the regulation of cGMP-PKGIα, Nrf-2-HO-1, and MAPK axes *in vitro*. They concluded that melatonin could ameliorate oxidative stress, reduce apoptosis, and restore cardiac function by regulating MAPK and Nrf-2-HO-1 axes in MI reperfusion injury in diabetic subjects. cGMP-PKGIα signaling coupled with membrane receptors, particularly MT_2_ receptors, plays a major role in this process (222).

Mitochondrial fission is a process by which the mitochondrial structure is initially fragmented into small particles during MI and/or reperfusion injury (223). Abundant mitochondrial fission results in damage to the mitochondrial DNA. These degraded mitochondria cannot generate adequate mitochondrial respiratory complexes, which results in enhanced synthesis of ROS and reduced oxidative phosphorylation (224). Furthermore, fragmented mitochondria release pro-apoptotic mediators such as cytochrome c into the cytoplasm, which triggers the mitochondrial apoptosis pathway (225). According to previous studies, reducing mitochondrial fission could ameliorate damage after MI and/or reperfusion injury. The opposite process of mitochondrial fusion can stimulate the mitochondria, and allow damaged mitochondria to repair themselves (226). The mitochondrial fusion molecule OPA1 (optic atrophy 1) is a GTPase involved in the repair of mitochondrial DNA, or the disposal of unrepairable mitochondria by the process of mitophagy (227). Maintenance of the OPA1 level interferes with the process of mitochondrial fission, leading to the suppression of reperfusion injury in the brain (228) and the liver (229). Likewise, a cardiac reperfusion model suggested that upregulation of OPA1 could maintain the viability of cardiac muscle cells and provide mitochondrial homeostasis (230). The inhibitory activity of melatonin against mitochondrial fission has been reviewed in prevuious publications (231, 232). Nevertheless, the upstream mediators of OPA1-induced mitochondrial fusion remain poorly understood.

Yap is a main downstream regulator of the Hippo pathway, and has been shown to be involved in cardioprotection in myocardial reperfusion injury (233). High Yap levels suppress the expression of Mst1, which decreases reperfusion-induced apoptosis in cardiac muscle cells (234). The Yap–Hippo pathway can also inhibit mitochondrial fission mediated by Drp1 (dynamin-related protein 1) and thus ameliorate reperfusion injury in the heart or the brain (235). Up-regulation of Yap promotes mitochondrial autophagy and reduces mitochondrial fission (43). The cross-talk between Yap–Hippo signaling and mitochondrial fission has been shown in previous studies (236). Nonetheless, if Yap plays a role in mitochondrial fusion during reperfusion was uncertain.

In a study by Ma and Dong the beneficial effects melatonin on mitochondrial fusion induced by OPA1 were studied in MI and/or reperfusion injury (237). According to their study, melatonin could preserve myocardial function, decrease the infarct area and reduce death of cardiac myocytes in response to cardiac reperfusion stress. Melatonin increased expression of OPA1, which largely restored the mitochondrial fusion, which had been inhibited by MI or reperfusion. Silencing of OPA1 abrogated the protective effects of melatonin on mitochondrial apoptosis and mitochondrial energy metabolism. Furthermore, their study showed that melatonin could regulate expression of OPA1 *via* the Yap–Hippo pathway, inhibition of which resulted in death of cardiac muscle cells and damage to mitochondria despite the treatment with melatonin (237).

The JAK/STAT axis regulates several biological activities, such as proliferation, differentiation, tumor metastasis, and inflammation. Upregulating the JAK2/STAT3 axis can decrease apoptosis and oxidative stress in response to MI and/or reperfusion. Furthermore, the JAK2/STAT3 signaling pathway could be involved in cardiac protection in ischemia and/or reperfusion injury (238, 239). It still remains unclear whether the JAK2/STAT3 signaling pathway is involved in melatonin-induced cardiac protection in the heart.

The success of heart transplantation is critcally dependent on the satisfactory functioning of the donor heart when removed from the donor after circulatory death (DCD). Lan et al. developed a DCD heart model to identify the effects of melatonin on myocardial function in donor hearts, and investigated whether JAK2/STAT3 signaling was involved in the mechanism of action (240). Donor hearts were obtained from DCD model rats, which had received melatonin pre-treatment or not. They took biopsies 3, 12, and 24 h following heart transplantation. Myocardial edema was measured by the wet/dry ratio and water content, while hematoxylin and eosin staining assessed inflammation. Levels of expression of IL-6, TNF-α, and matrix metalloproteinase-9 were measured. Oxidative stress was quantified by the activity of GPx and SOD, MDA levels, and expression of cytochrome-C, Nrf2, and NQO1. Cellular apoptosis was determined by measuring Bcl-2, Bax, cleaved caspase-3, and total caspase-3. To assess JAK2/STAT3 activity, p-STAT3 and p-JAK2 levels were measured by Western blotting. Melatonin was shown to exert cardiac protection against MI and/or reperfusion by decreasing myocardial inflammation and edema, inhibiting apoptosis and oxidative stress, and activating the JAK2/STAT3 axis. The JAK inhinitor AG490 (tyrphostin) suppressed all of these effects. In conclusion, melatonin may protect cardiac muscle against reperfusion injury caused by *ex vivo* perfusion in a DCD heart transplant model by activating the JAK2/STAT3 signaling pathway (240).

Mitophagy is a way of eliminating defective mitochondria by destroying them inside lysosomes (241, 242). Two proteins, PINK1 (PTEN-induced kinase 1) and Parkin (E3 ubiquitin ligase) have been shown to control mitophagy (241, 243). Alternative (non-conventional) mitophagy may act to reduce ischemic stress in the heart, as suggested by recent research. A recent study showed that the proteins ULK1, Rab9, Rip1, and Drp-1 were involved in alternative mitophagy in cardiomyocytes under stress. This kind of mitophagy is thought to be the most common type during stressful conditions, and is distinctly different from PINK1-Parkin dependent mitophagy (244).

Melatonin helps in closing the mitochondrial permeability transition pores (MPTPs) (245, 246). When the subcellular distribution of melatonin was measured, it was found that the concentration inside the mitochondria was much higher than that measured in the blood (128, 247). Besides passive diffusion, melatonin is actively transported into mitochondria, which enhances cell tolerance to different insults (128, 248).

Aralkylamine N-acetyltransferase (AANAT) and acetylserotonin o-methyltransferase (ASMT) are two important enzymes involved in melatonin biosynthesis, which were recently found to be expressed in the mitochondrial matrix of mouse brain (249). The outer mitochondrial membrane contains the highly specific MT1 receptor, which has high affinity for melatonin. Adenylate cyclase activity is inhibited by melatonin, as is the release of cytochrome C in response to stressful conditions (250). More research is required to understand the importance of melatonin in the mitochondria, and whether they can take up or synthesize this hormone. Moreover, how melatonin affects mitochondrial dynamics under stressful conditions, and whether it can protect the heart from oxidative stress is uncertain.

Dube et al. (251) investigated how both conventional and non-conventional mitophagy could affect oxidative phosphorylation in rat hearts. The hearts were isolated and perfused for 30 min, then exposed to ischemia for 20 min, and then reperfused for another 30 min. Biopsy samples were used to assess mitochondrial oxygen consumption. Melatonin was administered before ischemia and after reperfusion. Melatonin had a minimal effect on mitochondrial O_2_ consumption, which was notably decreased after reperfusion. Beclin 1 was shown to be decreased by ischemia and increased after reperfusion, but in both states, PINK1 and Parkin were reduced. Reperfusion increased p62 expression. During myocardial IR, Rab9 activates a surrogate type of mitophagy. Hemorrhage lowered the cytosolic expression of ULK1, while reperfusion enhanced it, which was linked with Rab9 and Drp1 being redistributed from the mitochondria to the cytosol. Melatonin significantly reduced mitochondrial p62 expression in IR injury. Overall, melatonin I increased levels of ULK1, Rab9, and P-ULK1, while decreasing levels of pDrp1 as well as the mitochondrial P/t Drp1 ratio. This suggests that melatonin may be able to inhibit fission of mitochondria. Fusion was also affected, but to a lesser extent compared to the other processes. Although cardioprotection by melatonin is linked to its effects on mitophagy, the relevance of these findings has yet to be established (251).

Cardiac cell degeneration following IR injury significantly involves apoptosis (252, 253). The pan-caspase inhibitorz VAD is able to reduce cellular death triggered by reperfusion (254–257). Apoptosis and necroptosis are the primary modes of death following IR injury, according to these studies. On a molecular level, Ripk3 (receptor-interacting serine/threonine-protein kinase 3) activity can regulate necroptosis (258). Ripk3 stimulates PGAM5 (phosphoglycerate mutase 5) to open MPTPs, disturbs energy generation, and therefore can make the organelles or cells swell in size (259, 260). But how IR and necroptosis are connected at the microvascular level, and how Ripk3 affects MPTPs was unclear. Some clinical trials have used melatonin to reduce the size of the infarct after MI, however, its effect on microvascular protection has not been clearly shown (126, 261–264). How Melatonin could prevent endothelial damage by necroptosis, because earlier studies have shown that it can prolong microvascular blood flow, resulting in decreased endothelial damage during myocardial IR (232, 265).

In a study by Zhou et al. the researchers sought to determine how IR injury and necroptosis were connected at the microvascular level, and whether interactions between Ripk3, PGAM5, and mPTP could be inhibited by melatonin (266). It has been shown that Ripk3 is the primary mediator of microvessel barrier failure, endothelial necrosis, capillary hyperpermeability, and inflammatory response in IR injury. After the genetic deletion of Ripk3, myocardial IR damage, and the endothelial function was improved, and the same benefits were provided by melatonin, which inhibited Ripk3 and gave a pro-survival advantage during IR. Ripk3 upregulates PGAM5, which phosphorylates CypD, and causes the MPTPs to open thus leading the endothelial cells toward necroptosis. Melatonin was able to suppress this process resulting in reduced necroptosis. A novel route for IR-mediated microvascular damage and endothelial necroptosis was demonstrated by these studies, namely the Ripk3-PGAM5-CypD/mPTP axis. Melantoin therapy on the other hand decreased cellular necroptosis by inhibiting the Ripk3/PGAM5/CypD/mPTP cascade, and protecting the endothelial system under IR stress (266).

IR damage is caused by Ca2+ excess, which leads to cardiomyocyte death under these conditions. Melatonin may protect the heart from IR damage by regulating intracellular calcium homeostasis, although this is not completely understood. Myocardial damage caused by prolonged hypoxia may be protected by melatonin, according to Yeung et al. Melatonin is thought to enhance calcium handling in cardiomyocytes by an antioxidant mechanism (267). Ca2+ overload under acute IR stress has not been well-investigated, so the effects of melatonin and the underlying mechanism are not well-understood. Cellular intracellular calcium handling and cell death are mediated by the cardiac proteins IP3R and SERCA2a, both of which are involved in intracellular calcium handling, cell contractility, and cell death (268–271). A recent study has demonstrated that IR activates an anti-apoptotic pro-survival kinase mechanism, e.g., ERK1/2 (extracellular signal-regulated kinase) and p42/p44 (272, 273).

In their study Hu et al. sought to determine if melatonin could protect cardiomyocytes from IR injury by regulating IP3R (inositol triphosphate receptor) and SERCA2a (sarcoplasmic/endoplasmic reticulum Ca2+ ATPase 2a) to decrease calcium overload *via* the ERK1 pathway (274). H9C2 cells were used in an *in vitro* study to simulate hypoxia/reoxygenation (H/R). The actin filament organization in cardiomyocytes was assessed by phalloidin staining, while Fura-2/AM was used to assess intracellular Ca2+ levels. Using a myocardial ischemia/ reperfusion (I/R) paradigm in rats, immunofluorescence labeling was used to identify the expression of IP3R and SERCA2a in the myocardium. H/R cardiomyocytes treated with melatonin showed a reduction in calcium overload as well as a decrease in IP3R expression and an increase in SERCA2a expression, mediated via ERK1. These effects could be reversed by PD98059, a small molecule inhibitor of MEK1 activation and MAP kinase signaling. IP3R and SERCA2a can regulate ERK1 to maintain intracellular calcium concentration at a stable level. They concluded that melatonin-induced cardioprotection against IR injury was at least in part due to ERK1 (274).

Table 3 lists some studies on the therapeutic effects of melatonin on ischemia reperfusion injury.

**Table 3:** Therapeutic effects of melatonin against ischemia-reperfusion injury.

**Melatonin dose**	**Route of administration**	**I/R duration**	**Treatment duration**	**Target**	**Effect**	**Model**	**References**
10 mg/kg	Intraperitoneal	30 min/3 h, or 30 min/6 h	5 days	cGMP, PKG, Nrf-2-HO-1, MAPK	Ameliorated diabetic I/R injury, reduced cardiac cell apoptosis, and oxidative stress	*In vivo* (rats)	(222)
10 μM or 10 nM	Administered into perfusion solution	20/40 min	5 min before ischemia, also simultaneous with reperfusion	–	Decreased arrythmia and VF caused by reperfusion	*In vivo* (rats)	(275)
50 mg	Intraoperative	–	3 days	Troponin I (TpI)	Reduced cardiac damage	Humans	(261)
10 mg	Oral						
10 mg/kg	–	30/120 min	10 days	Fas, cytochrome b-245 beta chain (Cybb), irisin, nuclear factor-κB (Nf-κB)	Improved protective effect of remote ischemic preconditioning (RIPerC) against I/R injury	*In vivo* (rats)	(276)
50 mM	Added to reperfusion solution	30 min/60 min	20 min	ROS	Reduced mitochondrial oxidative stress, increased mitochondrial membrane potential	*In vivo* (rats)	(277)
10 mg/kg	Intravenous	30/120 min	10 min	GSH, MDA	Increased antioxidants in cardiac cells, inhibited lipid peroxidation	*In vivo* (rats)	(278)
2/5, 5, 10 mg/kg	Intraperitoneal	10/15 min	10 min	–	Inhibited myocardial apoptosis during IR, protected mitochondrial structure & function	*In vivo* (rats)	(279)
5 μM	–	12/12 h	12 h	ERK1, IP3R, SERCA2a	Inhibited cardiomyocyte apoptosis, improved actin filament organization	*In vitro*	(274)
20 mg/kg	Intraperitoneal	30 min/2 h	12 h	IP3R and SERCA2a	Induced cardioprotection against IR injury	*In vivo* (rats)	(274)
50 μM	Administered into perfusion medium	30/15 min	15 min	Cardiolipin	Improved cardiac cell viability by keeping MPTPs closed	*In vivo* (rats)	(280)
6 mg/kg	Intraperitoneal	30/30 min	3 weeks	TBARS, MDA	Prevented microvascular injury & ventricular arrhythmia	*In vivo* (hamsters)	(281)
50 mg/kg	Intraperitoneal	20/20 min	30 min	Troponin T (cTn-T), MDA, SOD, myeloperoxidase (MPO)	Protected against cardiac IR injury	*In vivo* (rats)	(282)
20 mg/kg	Intraperitoneal	30 min/2 h	12 h	Beclin 1, LC3-II, AMPK, mTOR	Protected CMECs against IRI by inhibiting autophagy	*In vivo* (rats)	(283)
5, 10, 20, 50 μM	Melatonin added to perfusion solution	10 min/–	10 min	–	Decreased arrhythmia, suppressed oxidative damage	*In vivo* (rats)	(284)
50 μM	Administered into perfusion medium	30/45 min	3 min	MDA	Significantly suppressed of apoptosis	*In vivo* (rats)	(285)
10 mg/d 20 mg/d	Oral	–	5 days	Troponin-I, IL-1β, iNOS, caspase-3	Suppressed IR damage	Humans	(262)
10 mg/kg	Intraperitoneal	30 min/3 h	5 days	Sirt3	Protected against IR injury, alleviated myocardial oxidative stress	*In vivo* (mice)	(286)
50 μM	–	35/30 or 120 min	40 min	p38MAPK, ERK, PKB/Akt, NOS, guanylyl cyclase	Cardioprotective effects, anti-adrenergic activity	*In vivo* (rats)	(36)
10 mg/kg 20 mg/kg	Intraperitoneal	45 min/4 h	24 h	OPA1, LDH, CK-MB, troponin T, TNFα, IL-6, and MCP1	Promoted mitochondrial fusion, restored energy generation, prevented myocardial IR injury	*In vivo* (rats)	(287)
10, 20 μM	–	45 min/ 4 h	24 h	OPA1	Reversed IR-mediated myocardial dysfunction	*In vitro*	(287)
0.025 μg/h	Infusion into hypothalamic paraventricular nucleus (PVN)	30 min/6 h	1 week	Cu/Zn-SOD, NOX2, NOX4, IL-1b, NF-kB, p65, IL-10	Modulated oxidative stress & inflammatory pathways in PVN, reduced myocardial IR injury	*In vivo* (rats)	(288)
10 mg/kg	Intraperitoneal	15/30 min	7 days	MMP-9, IL-6, TNF-α, MDA, SOD, GPx, Nrf2, NQO1, cytochrome-C, Bax, Bcl-2, caspase-3, p-JAK2, p-STAT3	Decreased edema, inflammation, oxidative damage, & apoptosis in cardiomyocytes	*In vivo* (rats)	(240)
20 mg/kg			30 min				
0.3, 50 μM	–	20/30 min	10 + 10 min	Cytosolic beclin 1, LC3 II/I, p62, ULK1, Rab9, Drp1	Prevented mitochondrial fission, modulated non-conventional mitophagy, promoted autophagy	*In vivo* (rats)	(251)
50 μM	Melatonin added to perfusion solution	30/15 min	15 min	–	Preserved mitochondrial complexes I, III, cardiolipin from oxidative damage	*In vivo* (rats)	(246)
0.3 mM	Melatonin and HTK (histidine, tryptophan, ketoglutarate) added to Tyrode's solution	80/45 min	30 min	ROS	Attenuated postischemic ROS burst, but was unable to improve the functional recovery provided by HTK	*In vivo* (guinea pigs)	(289)
20 mg/kg	Intraperitoneal	45 min/4 h	24 h	OPA1	Protected cardiac function, increased survival	*In vivo* (mice)	(290)
5 μM	–	45 min/4 h	12 h	OPA1	Reversed loss of MMP, restored energy production in cardiac cells	*In vitro*	(290)
20 mg/kg	Intraperitoneal	30 min/3 h	10 min	Sirtuin-3 (SIRT3), lactate dehydrogenase	Improved post-ischemic cardiac structure & function, decreased apoptosis, & oxidative damage	*In vivo* (mice)	(291)
20 mg/kg	Intraperitoneal	45 min/6 h	12 h	Ripk3-PGAM5-CypD-MPTP cascade, LDH, troponin T, CK-MB	Decreased endothelial necroptosis	*In vivo* (mice)	(266)
20 mg/kg	Intraperitoneal	2/4 h	12 h	PPARγ	Suppressed HR damage, inhibited platelet activation, reduced FUNDC1 (FUN14 domain-containing protein 1) mediated mitophagy	*In vivo* (mice)	(292)
12 mg/kg/d	Intraperitoneal	30 min/4 h, 30 min/6 h, 30 min/72 h	3 days+15 min	PERK, eIF2α, ATF4, RISK, SAFE pathway, ERK1/2 pathway	Reduced myocardial apoptosis & oxidative stress, improved cardiac function	*In vivo* (mice)	(293)
20 mg/kg/d	Oral	30 min/4 h, 30 min/6 h, 30 min/72 h	1 week	MDA, SOD, PERK, eIF2a, ATF4, CHOP, SIRT1	Ameliorated reperfusion-induced oxidative & ER stress, reduced MI/R damage, improved cardiac function	*In vivo* (rats)	(294)
5 μM	Melatonin added to perfusion solution	45/60 min	5 min	JAK2/STAT3 signaling pathway, SOD, H2O2, MDA, LDH, GSH	Melatonin pretreatment attenuated IR injury, reduced mitochondrial oxidative damage	*In vivo* (rats)	(295)

## Melatonin and Blood Pressure: Recent Evidence and Signaling Pathways

Nitric oxide (NO) has protective effects on the cardiovascular system, and shows antiproliferative, antifibrotic, and antihypertensive activity (296, 297). Prolonged administration of L-NAME (Nγ-nitro-L-arginine methyl ester) to rats inhibits NO synthesis and release, causing organ damage and hypertension (298–300). Another effect of L-NAME is to weaken the renal artery by affecting RAAS (renin–angiotensin–aldosterone system) and promoting renin release (295, 296).

When rats were subjected to continuous light exposure and experimental pinealectomy, the resulting melatonin deficiency led to myocardial fibrosis and hypertension (301, 302). Melatonin has pleiotropic effects *via* nuclear receptors as well as membrane receptors (303, 304). Furthermore, it provides cardiovascular protection by ROS scavenging, and endothelial protection *via* sympatholytic effects (19, 25, 126, 301, 305, 306). However, how melatonin affects neurohumoral pathways, including the RAAS was not clear.

Simko et al. evaluated the structural and hemodynamic effects of L-NAME and its connection with the RAAS, and how melatonin could benefit them (307). Wistar rats were divided into 4 groups. The first group received melatonin, the second group received L-NAME, the third group received l-NAME + melatonin, and the fourth group was an untreated control group. Hypertension and LV fibrosis were quantified by measuring soluble, insoluble, and total collagen levels. Melatonin led to a decrease in the amount of total and insoluble collagen in the left ventricle, and also lowered systolic blood pressure. L-NAME decreased serum angiotensin II (Ang2) and its derivatives, but these they were unaltered by melatonin. The L-NAME group showed elevated serum aldosterone as well as increased aldosterone to Ang2 ratio (AA2-ratio), while the melatonin group showed no change in these two measures. In conclusion, L-NAME exerts its hypertensive effects through lowering Ang II and increasing aldosterone, while melatonin reverses hypertension without modifying the RAAS (307).

The most common treatment for hypertension involves a constant drug dosage, which ignores the daily cycle and rhythm of blood pressure. Hermida et al. showed that antihypertensive medication that takes into account the body's natural rhythms and cycles was more effective compared to traditional blood pressure treatment. This regimen provided a bigger reduction in hemorrhagic and ischemic strokes, fewer myocardial infarctions, and reduced cardiovascular death (308). Because the RAAS is active during sleep, antihypertensive medication during the night has more benefits and reduces cardiovascular complications (308, 309). The results revealed that blood pressure treatment without taking into account the circadian rhythm was not so successful; therefore, physicians should consider antihypertensive chronotherapy. Different types of brain hormones, such as melatonin, must synchronize with the body clock in order to maintain appropriate blood pressure (310, 311). Two additional characteristics of melatonin make this neurohormone suitable for protecting against hypertension, its antioxidant and anti-inflammatory properties (312–314). Pechanova et al. discussed the peripheral and central effects of melatonin on blood pressure regulation, highlighting the fact that melatonin reduced inflammation, oxidative damage, and promoted endothelium function (34). The effects of melatonin on blood pressure may involve the modulation of nitric oxide (NO), angiotensin II (Ang II), and endothelin (ET) (315–317). When human umbilical vein endothelial cells (HUVEC) were treated with 1-palmitoyl-2-(5-oxovaleroyl)-sn-glycero-3-phosphocholine (POVPC), a product of oxidized low-density lipoprotein that acts as a proinflammatory lipid, they developed a deficiency in NO production and eNOS activity, as typically found in atherosclerosis (318). In a HUVEC model, melatonin decreased angiogenesis triggered by VEGF (319).

Shao et al. also used HUVECs to assess how melatonin affected endothelial function, circadian changes in blood pressure and hypertension, and also the molecular mechainism (320). To simulate hypertension, they incubated HUVECs under 25 kPa external pressure, and incubated them with melatonin. They measured vasoactive agents, namely Ang II, endothelin (ET), eNOS, and NO. Melatonin significantly reduced endothelin at 18 and 24 h and angiotansin II at 18 h after treatment, and led to a rise in NO levels and elevated eNOS activity at 6–12–18 and 24 h. Melatonin could regulate genes related to circadian rhythm, cGMP-PKG activity, NO production, and renin/insulin metabolism, possibly explaining its effects on blood pressure. In conclusion, melatonin exerts its circadian protective effects on hypertension by lowering Ag II and ET, and elevating NO and eNOS (320).

Melatonin, in addition to its antioxidant properties, appears to play a role in epigenetic regulation, according to recent research (321). Epigenetic modification mediated by melatonin could prevent cellular programmes that affect hypertension (322, 323). The effects of melatonin on programming complications related to endangered pregnancies have been recognized (324), but how it affects programmed hypertension over long periods is unclear. However, some theories have been suggested, such as corticosteroid effects, oxidative damage, epigenetic modification, RASS modification, and reversing the loss of nephrons in the kidney (325).

Tain et al. investigated if high fructose (HF) consumption by the mother could cause programmed hypertension, and if melatonin was able to protect against this process through epigenetic regulation (326). During pregnancy and lactation, Sprague-Dawley rats were fed ordinary chow, chow + HF, or chow + HF + melatonin. HF comsumption by the mother increased the blood pressure in the 12-week old offspring. Melatonin inhibited this process by increasing kidney levels of NO. Melatonin downregulated SEH (soluble epoxide hydrolase) a gene which is involved in blood pressure regulation. In addition, they found that there were some genes involved in arachidonic acid metabolism that may mediate hypertension triggered by HF, and melatonin could regulate them. Their results suggested that melatonin can increase NO levels in the kidney, inhibit SEH expression, and epigenetically modulate blood pressure-controlling genes (326). eNOS and ADMA (asymmetric dimethylarginine) are able to induce hypertension by inducing oxidative stress (327). In rats born from mothers with malnutrition/diabetes, a defective ADMA-NO axis and a reduction in nephrons were linked to hypertension (328, 329). In addition, a low protein diet may cause hypertension through epigenetic changes affecting RASS (330). HDAC1-3 is widely expressed in nephron precursors, and HDAC enzymes can affect nephron generation (331). In a rat model, melatonin treatment inhibited oxidative stress as well as reducing hypertension (332). It also prevented Ang II induced hypertension (333). New data has clarified how melatonin could epigenetically modulate HDACs (321, 334, 335). Melatonin has a protective role in the rat placenta against oxidative/nitrosative mitochondrial damage and IR injury (336). Melatonin can pass through the placenta during pregnancy where it plays a key role in fetal development, but its epigenetic activity needs more research (324).

Tain et al. assessed the protective effects of melatonin on programmed hypertension triggered by corticosteroids during pregnancy (322). They divided young rats into four groups: (1) control; (2) dexamethasone (DEX); (3) control plus melatonin; (4) DEX plus melatonin. Pregnant rats were administered with the above agents, and hypertension occurred in group 2 at week 16, which was decreased by melatonin in group 4. If the nephrons were reduced, DEX will accumulate in the kidneys, which could be inhibited by melatonin therapy. All groups had equal kidney contents of superoxide and NO. DEX upregulated prorenin and renin receptors, as well as histone deacetylase-1 (HDAC-1) in the kidneys of 4-month old rats. Melatonin increased the weight of the kidneys in group 4, and upregulated HDAC-1, HDAC-2, and HDAC-8 in the kidneys of groups 3 and 4. Melatonin could inhibit hypertension triggered by DEX during pregnancy, by altering RAS components, protecting nephrons, and regulating HDACs (322).

Chronic kidney disease (CKD) is accompanied by high blood pressure when NO production is reduced (337, 338). Endogenous inhibitors of NOS, such as ADMA can lead to a decrease in NO synthesis. CKD and high ADMA levels are often seen in patients with hypertension (327). Many experimental models have been used to produce renal failure leading to hypertension. As spontaneously hypertensive rats (SHR) get older, the onset of CKD is an inevitable consequence (339). Mature SHRs treated with L-NAME, a NOS inhibitor, developed accelerated glomerulosclerosis as well as premature kidney failure (340). It was shown that melatonin produced by the pineal gland prevents the ADMA level from rising, and reduces the blood pressure in young SHRs (332). Melatonin may suppress hypertension by upregulating DDAH (an enzyme that degrades ADMA) in the kidneys of SHRs (332). It has also been shown that a reduction in renal ADMA concentrations may be protective against hypertension (341). However, whether L-NAME could induce nephrosclerosis in mature SHRs, or even in young SHRs was uncertain (327).

Young SHRs were used by Cheng and colleagues to investigate the interactions between L-NAME, ADMA, and melatonin (312). They randomly allocated 4-week old SHR rats into three groups: (1) control; (2) L-NAME; (3) L-NAME + melatonin. Rats were sacrificed at 10 weeks old. L-NAME caused renal dysfunction, glomerular sclerosis, and high blood pressure in young SHRs. L-NAME led to a lower arginine-to-ADMA ratio, but increased the total level of ADMA, but melatonin could reverse these effects. The researchers were able to restore DDAH (dimethylarginine dimethylaminohydrolase) activity with melatonin treatment. Melatonin therefore led to a decrease in ADMA concentration, restored the production of NO, increased the arginne-to-ADMA ratio, and decreased the amount of 8-hydroxydeoxyguanosine immunostaining (a marker of DNA oxidative damage) in SHR kidneys treated with L-NAME (310). They also proved that young SHRs were amenable to L-NAME effects.

Table 4 lists some studies on the effects of melatonin on high blood pressure.

**Table 4:** Effects of melatonin and blood pressure.

**Drug or pharmacological agent**	**Melatonin dose**	**Treatment duration**	**Targets**	**Effects**	**Model**	**Disease or condition**	**References**
Melatonin	–	–	–	Ameliorated hypertension in pregnant mice	*In vivo* (mice)	Gestational hypertension	(342)
Melatonin	6 mg/d	8 weeks	–	Controlled blood pressure	Humans	Type 2 diabetes mellitus	(343)
Melatonin	10 mg/kg/d	11 days	Antioxidant capacity, plasma MDA, sFlt-1, Nrf2, PlGF, HO-1	Markedly lowered blood pressure	*In vivo* (rats)	L-NAME-associated pre-eclampsia	(344)
Melatonin	6 mg/d	12 weeks	–	Improved blood pressure	Humans	Non-alcoholic fatty liver disease (NAFLD)	(345)
Melatonin	30 mg/kg/d	15 days	ROS	Effectively reduced baseline MAP	*In vivo* (rats)	Neurogenic hypertension	(346)
Melatonin	24 mg/d	4 weeks	–	No statistical effect on nighttime or daytime systolic or diastolic blood pressure	Humans	Essential hypertension	(347)
Melatonin	10 mg/kg/day	4 weeks	Renin-angiotensin-aldosterone system (RAAS)	Reduced systolic blood pressure	*In vivo* (rats)	L-NAME-induced hypertension	(307)
Melatonin	10 mg/d	12 weeks	NO, MDA, protein carbonyls (PCO), HDL-cholesterol, hs-CRP	Improved blood pressure	Humans	Type 2 diabetes & CAD	(348)
Melatonin	5 mg/kg/d	20 days	TNF-a, IL-6, VEGF, sFlt-1	Decreased blood pressure	*In vivo* (rats)	Pre-eclampsia	(349)
Melatonin	10 mg/kg/d	6 weeks	NOS, NF-κB	Had no effect on SBP	*In vivo* (rats)	Lactacystin-induced hypertension	(350)
Melatonin	5 mg/kg/d	3 weeks	KCNQ & KCNH2 genes	Prevented increase in blood pressure	*In vivo* (rats)	Pinealectomy & L-NAME-induced hypertension	(351)
Melatonin	10 μM	6, 12, 18, 24 h	Endothelin, Ang II, NO, eNOS	Circadian antihypertensive effects	*In vitro* (HUVECs)	Hypertension	(320)
Melatonin	10 mg/kg/d	3 weeks	eNOS & nNOS protein expression	Decreased blood pressure	*In vivo* (rats)	Metabolic syndrome	(314)
Melatonin	6 mg		–	Lowered systolic, diastolic & mean blood pressure	Humans	Laryngoscopy & endotracheal intubation	(352)
Melatonin	5 mg/kg		Cortisol, Ang I, Ang II, aldosterone, ANP, CRH, ACTH, endothelin	Reduced fetal hypertension	*In vivo* (sheep)	Fetal blood pressure	(317)
Melatonin	5 mg/kg/d	6 weeks	MDA, uric acid, renal aquaporin-3 (AQP-3)	Decreased systolic blood pressure	*In vivo* (rats)	Metabolic syndrome	(353)
Melatonin	1.5 mg/d	2 weeks	–	Reduced SBP & DBP levels	Humans	Elderly	(354)
Melatonin	3, 5 mg/d	8 weeks	-	Regulated blood pressure in circadian rhythm, reduced nocturnal hypertension	Humans	Type 2 diabetes & hypertension	(355)
Melatonin	0.01% melatonin in drinking water	Entire duration of pregnancy & lactation	Ephx2, Col1a2, Gucy1a3, Npr3, Aqp2, Hba-a2, Ptgs1 genes, NO, soluble epoxide hydrolase (SEH)	Blunted maternal high fructose (HF)-induced hypertension	*In vivo* (rats)	Maternal HF-induced hypertension	(326)
Melatonin	0.01% melatonin in drinking water	Entire duration of pregnancy & lactation	Renal superoxide, NO, renin–angiotensin system, Mas protein, histone deacetylase (HDAC)-1, HDAC-2, HDAC-8	Attenuated prenatal DEX induced hypertension	*In vivo* (rats)	Dexamethasone-induced hypertension	(322)
Melatonin	10 mg/kg/d	6 weeks	–	Partially prevented increased systolic blood pressure	*In vivo* (rats)	Continuous light-induced hypertension	(356)
Melatonin	25 μg/mL in drinking water	10 weeks	Hypophysial-testicular axis	Significantly blunted SBP	*In vivo* (rats)	Metabolic syndrome	(357)
Melatonin	0.01% melatonin in drinking water	6 weeks	Asymmetric dimethylarginine (ADMA), arginine, dimethylarginine dimethylaminohydrolase (DDAH), NO, 8-hydroxydeoxyguanosine	Lowered blood pressure	*In vivo* (rats)	Spontaneous hypertension + L-NAME	(312)
Piromelatine (melatonin agonist)	5, 15, 50 mg/kg/d	5 weeks	Plasma glucose, insulin, triglyceride, adiponectin, total cholesterol, HDL & LDL/VLDL cholesterol	Reduced blood pressure	*In vivo* (rats)	Spontaneous hypertension	(358)
Melatonin	10 mg/kg/d						
Melatonin	10 mg/kg/d	4 weeks	Oxidative stress	Prevented doxorubicin-induced increase in systolic blood pressure	*In vivo* (rats)	Doxorubicin-induced nephrotoxicity	(359)
Melatonin	10 mg/kg/d	Beginning of pregnancy up to 3rd week postpartum	Renal GPx, glutathione s-transferase (GST), total glutathione, SOD, catalase, glutathione reductase	Lowered systolic blood pressure, delayed but not completely eliminated hypertention	*In vivo* (rats)	Spontaneous hypertension	(360)
		After weaning until 16 weeks					
Melatonin	5 mg/d	2 months	Glucose, serum lipids, C-reactive protein, fibrinogen, catalase, GPx, SOD, TBARS	Lowered blood pressure	Humans	Metabolic syndrome	(361)
Melatonin	10 mg/kg	5 weeks	Conjugated dienes, NOS, COX-1, COX-2	Slight antihypertensive effect	*In vivo* (rats)	L-NAME-induced hypertension	(362)
Melatonin	5 mg/d	90 days	–	Decreased nocturnal blood pressure, increased daytime blood pressure	Humans	Coronary artery disease, circadian hypertension	(363)
Melatonin	10 mg/kg/d	5 weeks	NOS, eNOS, NF-κB, conjugated dienes, collagenous proteins, hydroxyproline	Reduced systolic blood pressure	*In vivo* (rats)	Spontaneously hypertensive rats	(364)
Melatonin	1 mg/kg/d	15 days	Angiotensin II, GABA(A) receptors	Prevented blood pressure increase, reduced blood pressure in developed hypertension	*In vivo* (rats)	Stress-induced hypertension	(365)
Melatonin	0.1 μL of 0.1 or 1.0 mM	14 days	Glutamate, GABA, taurine, MT1, MT2, and MT3	Reduced blood pressure	*In vivo* (rats)	Stress-induced hypertension	(366)
Melatonin	2 mg/d	4 weeks	–	Reduced nocturnal systolic blood pressure	Humans	Nocturnal hypertension	(367)
Melatonin	10 mg/kg	5 days	–	Reduced blood pressure	*In vivo* (rats)	L-NAME-induced hypertension	(368)
Melatonin	3 mg/d	3 weeks	–	Reduced nocturnal blood pressure	Humans (women)	Essential hypertension	(369)
Melatonin	10 mg/d	7 days	–	Lowered blood pressure	Humans	Type 1 diabetes	(370)
Melatonin	5 mg/d	1 week	–	Reduced nocturnal diastolic blood pressure	Humans	Type 1 diabetes	(371)
Melatonin	2.5 mg/d	3 weeks	–	Reduced nocturnal blood pressure	Humans	Essential hypertension	(88)
Melatonin	0.1 μL of 0.1 or 1.0 mM	15 days	ML1, ML2 receptors	Reduced mean arterial pressure	*In vivo* (rats)	Stress-induced hypertension	(372)
Melatonin	2.5, 5 mg/kg		–	Did not affect blood pressure	*In vivo* (rats)	–	(373)
Melatonin	1 mg	2 days	–	Reduced blood pressure	Humans (men)	–	(374)
Melatonin	10, 50 ng/kg		–	Reduced blood pressure	*In vivo* (Atlantic cod)	–	(375)
Melatonin	5 mg/d	4 weeks	–	Reduced blood pressure	Humans	Normotensive	(376)

## Effects of Melatonin on Atherosclerotic Plaque

Atherosclerosis-related cardiocerebrovascular diseases include, peripheral vascular disease (PVD), stroke, acute coronary syndrome (ACS), and stable angina pectoris (375). Atherosclerosis (AS) is characterized by the subendothelial accumulation of plaques in arteries, consisting of oxidized-LDL, and inflammatory cells like macrophages, T lymphocytes, and DCs (dendritic cells) (377, 378). The pathology starts with gradually progressive endothelial damage, which induces initial vascular lesions, eventually leading to the rupture of vulnerable plaque and the formation of thrombosis (379). Pyroptosis plays a crucial role in the pathophysiology of atherosclerosis, according to recent research (380). Pyroptosis is distinct from apoptotic cell death, instead representing a form of highly inflammatory necrotic cell death, wherein the plasma membrane ruptures and inflammatory factors such as interleukin (IL)-1β and IL-18 are released, along with other components from the cytoplasm (381).

Smoking can cause both inflammation and oxidative stress, thereby damaging the endothelial function (382). However, there has been little research into the relationship between smoking-induced oxidative stress and pyroptosis. A recent study suggested that nicotine could induce endothelial cell pyroptosis *via* the ROS/NLRP3 axis (383), and that the ROS pathway may interact with the pyroptosis-related pathway in inflammatory signaling.

Nrf2 acts as the major regulator of antioxidant enzymes like HO-1 (384). The Nrf2 pathway is activate under stressful conditions, and it is essential for sensing oxidative stress to protect cells against ROS (385). Nrf2 may have beneficial roles in cardiac IR damage (386), sepsis (387), and neurodegenerative diseases (388). Furthermore, Nrf2 has been shown to attenuate inflammation in smoking-induced chronic obstructive pulmonary disease, emphysema, and asthma (389, 390). However, the involvement of Nrf2 in smoking-induced vascular endothelial injury and its mechanism was unclear.

Zhao et al. investigated the interactions between melatonin and cigarette smoke in vascular injury (391). Cigarette smoke extract (CSE) could cause human aortic endothelial cells (HAECs) to undergo pyroptosis by affecting NLR Family Pyrin Domain Containing 3 (NLRP3). Furthermore, HAECs increased ROS production and Nrf2 activity in response to CSE. Nrf2-specific siRNA as well as an Nrf2 inhibitor were able to prevent CSE from activating the ROS/NLRP3 axis. In addition, Nrf2 increased cell survival and upregulated VEGF and eNOS. Melatonin suppressed intimal hyperplasia in a model of carotid artery injury. Melatonin also upregulated Nrf2, while suppressing the ROS/NLRP3 axis. In conclusion, melatonin could suppress atherosclerosis triggered by cigarette smoke by affecting the Nrf2/ROS/NLRP3 axis (391).

Macrophages are an important contributor to AS (392, 393). In the atheromatous plaque, they phagocytize oxidative LDL (ox-LDL) and form a necrotic core; they also secrete many pro-inflammatory mediators, resulting in degenerative and fibrotic changes, which increases the plaque size while reducing its stability, exposing it to rupture and thrombus formation (392). Melatonin was discovered to suppress ox-LDL modification *in vitro*, which may translate into less production of atherogenic plaques *in vivo*. Melatonin may also increase the plaque stability (393, 394). One study has been conducted to investigate whether melatonin could ameliorate vascular endothelial dysfunction, inflammation, and AS by inhibiting the Toll-like receptor 4 (TLR4)/nuclear factor kappa B (NF-κB) pathway in high-fat-fed rabbits (377). In this study rabbits were randomly divided into three groups: a standard diet (control group), a high-cholesterol diet (atherosclerosis group), and a high-cholesterol diet plus 10 mg/kg/day melatonin (melatonin group) for 12 weeks. When compared to the control group, a high-fat diet dramatically elevated serum lipid and inflammatory markers in rabbits with atherosclerosis. Results revealed that melatonin improves lipid metabolism, vascular endothelial dysfunction, and inflammation, as well as slowing the progression of atherosclerosis in high-fat-fed rabbits. Furthermore, it suggests that suppressing the TLR4/NF-κB system in local vasculature with atherosclerotic damage is critical for melatonin's protective effects (377). Endothelial dysfunction is linked to cholesterol feeding. Pita et al. (395) demonstrated that long-term administration of melatonin altered the fatty acid content of rat plasma and reduced fatty infiltration in the intima caused by cholesterol feeding. A research group shown that melatonin administration prevents *in vitro* smooth muscle cell inflammation and proliferation, as well as atherosclerosis in apolipoprotein E-deficient mice (396). Hepatocyte growth factor (HGF) may also exert beneficial effects in the cardiovascular system (397). Considerable evidence has recently been provided to show that HGF acts as a potent anti-inflammatory agent (377, 396–406). Cardiovascular pathology and AS have been observed to be associated with reduced local amounts of HGF in cardiac and vascular cells (398–400). Several drugs such as ARBs (angiotensin II receptor blockers) (400–402), ACEIs (angiotensin-converting-enzyme inhibitors) (399, 400, 403) and PPAR-γ agonists (404) have been shown to upregulated HGF, resulting in suppression of AS (402, 405). Interestingly, melatonin has also been shown to upregulate HGF expression both *in vitro* (189, 190) and *in vivo* (407).

The ability of melatonin to prevent macrophage infiltration and improve plaque stability by activating the HGF/c-Met (HGF receptor) axis was assessed by Hu et al. in rabbits, using USPIO-enhanced MRI to monitor AS plaques (406). They randomly assigned rabbits into three groups: (1) standard diet; (2) high-fat diet; (3) high-fat diet + melatonin. In the atherosclerotic abdominal aorta, melatonin notably reduced the signal voids in 3D-TOF MRI, decreased the standard signal intensity in T2WI MRI, and decreased the aortic luminal area in 2D-TOF MRI. Furthermore, melatonin reduced serum IL-6, intima/media thickness ratio, and CD68^+^ as well as USPIO-positive regions of the intima. Melatonin increased serum IL-10, HGF, and c-Met, and induced smooth muscle cells and collagen fibers to accumulate in the intima. In conclusion, melatonin notably prevented macrophage infiltration in the plaque, and its increased stability could be partially attributed to the HGF/c-Met axis (406).

The nuclear receptor RORα can regulate circadian rhythm, immune response, and cellular metabolism (408, 409). It has been proposed that some melatonin effects, like its anti-inflammatory activity could be attributed to RORα (410–412). In addition, some cardiovascular benefits of melatonin have been proposed to be mediated by RORα (42, 303, 413).

How melatonin affects atheromatous plaque and whether it was mediated by RORα was investigated by Ding et al. (393). They used ApoE^−/−^ mice with high cholesterol and elevated blood pressure to assess plaque vulnerability to rupture. The rate of plaque rupture was markedly reduced by melatonin. Melatonin suppressed inflammation inside the plaque by preventing plaque macrophages from differentiating into the M1 phenotype, by affecting RORα. Additional evidence has supported the fact that melatonin can modify the macrophage phenotype through RORα and affecting the AMPKα-STATs axis (393).

Collagen metabolism is regulated by the P4H (prolyl 4-hydroxylase) enzyme (407). The P4H α subunit (P4Ha1) converts procollagen into a mature and stable form of collagen (414). P4Ha1 inhibition led to a reduction in mature collagen, which reduced plaque stability (415).

Li et al. used ApoE^−/−^ mice to assess how melatonin affected plaque stabilization (394). Melatonin upregulated P4Hα1 expression in leiomyocytes *in vitro* by phosphorylating Akt and activating Sp1. This effect was blocked by small molecule inhibitors LY294002 (Akt inhibitor) or MTM (mithramycin a Sp1 inhibitor). Furthermore, melatonin stabilized plaque *in vivo* by upregulating P4Hα1, and MTM blocked this effect (394).

Table 5 lists some studies on the effects of melatonin on atherosclerosis and intimal hyperplasia.

**Table 5:** The effects of melatonin on atherosclerosis and intimal hyperplasia.

**Drug or pharmacological agent**	**Melatonin dose**	**Treatment duration**	**Targets**	**Effects**	**Model**	**References**
Melatonin	10 mg/kg/d	4 weeks	VEGF, eNOS, Nrf2/ROS/NLRP3 signaling pathway	Reduced rat carotid artery intimal hyperplasia, attenuated smoking-induced atherosclerosis	*In vivo* (rats)	(391)
Melatonin	5, 10 mg/kg/d	2 weeks	Vaspin, visfatin, DDAH, STAT-3	Protected against atherosclerosis, anti-inflammatory effects	*In vivo* (rats)	(416)
Melatonin	10 mg/kg/d	12 weeks	HGF/c-Met axis	Reduced number of macrophages in plaque, increased stability	*In vivo* (rabbits)	(406)
Melatonin	10 mg/kg/d	9 weeks	RORα, AMPKα-STAT pathway	Regulated plaque inflammation, increased plaque stability	*In vivo* (mice)	(393)
Melatonin	0.3, 3, 30 mg/kg/d	8 weeks	P4Hα1, Akt, Sp1	Stabilized plaque	*In vitro, in vivo* (mice)	(394)
Melatonin	10 mg/kg/d	7, 15 weeks	TNF-α, PDGF-BB	Suppressed atherosclerosis	*In vitro, in vivo* (mice)	(396)
Melatonin	20 mg/kg/d	4 weeks	NLRP3, Sirt3/FOXO3a/Parkin signaling pathway	Inhibited progression of atherosclerosis	*In vitro, in vivo* (mice)	(417)
Melatonin	20 mg/kg/d	4 weeks	Myosin light chain kinase (MLCK), ERK, JNK, p38	Inhibited atherosclerosis	*In vivo* (rabbits)	(418)
Melatonin	10 mg/kg/d	12 weeks	TLR4, MyD88, NF-κB, p65, IκB	Improved endothelial function, suppressed plaque formation	*In vivo* (rabbits)	(377)
DTBHB	0.02% wt/wt	16-weeks	IL-6, TNFa	Did not modify atherosclerosis	*In vivo* (mice)	(419)
Melatonin	0.02% (w/w)	16 weeks	–	Increased atherosclerosis	*In vivo* (mice)	(420)

## Melatonin and Cardiac Arrhythmia

Cardiac arrhythmias may lead to many complications including death (421). VT (ventricular tachycardia) and ventricular fibrillation (VF) commonly occur after cardiac IR injury, which may be fatal if untreated. Appropriate antiarrhythmic agents may prevent these outcomes. Oxidative stress occurring during IR injury may explain the onset of cardiac rhythm abnormalities. The “Metabolic sink” hypothesis (422–424) is as follows: superoxide anions pass through the mitochondrial inner membrane which induces a drop in the Δψm (MMP) and a reduction in cellular ATP levels. In response to these effects the sarcolemmal ATP-sensitive potassium current (IKATP) is increased. As voltage-gated potassium channels open to repolarize the membrane, the potassium conductance increases dramatically to bring the membrane potential closer to the equilibrium potential for potassium. ROS may also inhibit the sodium current (INa) to cause rhythm abnormalities (425). Melatonin ameliorates the shortened action potential, and upregulates connexin 43 during ischemia (426), which may explain its antiarrhythmic activity, along with its antioxidant effects (18). However, further resaerch is needed to confirm the relationship between melatonin, oxidative stress and cardiac rhythm abnormalities.

Sedova et al. used an IR injury model to assess how VT and VF, oxidative damage, cardiac electrophysiological parameters, and melatonin may be connected to each other (427). Melatonin reduced the rate of VT and VF, shortened baseline activation times (ATs), as well as activation-repolarization intervals, and also improved recovery of repolarization times (RTs). SOD activity was observed to be elevated in the melatonin group. *In vitro*, melatonin restored the action and resting membrane potentials even more In conclusion, melatonin affected repolarization through exerting antioxidant effects, while its suppression of arrhythmia could be attributed to its ability to improve ventricular function (427).

The anti-arrhythmic effects of melatonin could be attributed to its effects on action potential length (284, 428). Melatonin may suppress VF (426) by upregulating connexin-43 in the myocardium, which is the major mediator of electrical coupling. The probability that myocardium upregulates connexin-43 in acute hypokalemia is low, however melatonin may enhance the coupling between cells (429, 430).

Prado et al. investigated whether melatonin was able to suppress arrhythmia triggered by hypokalemia (431). A hypokalemic medium was employed, and melatonin and its receptor antagonist, luzindole, were added. They measured the connexin-43 concentration, and how it was dephosphorylated and distributed. Melatonin suppressed VF development, induced a delay in its development, as well as restoring potassium currents and accelerated sinus rhythm. Melatonin prevented the widening of the QRS, accelerated the development of action potentials, and shortened their length. Melatonin also inhibited the dephosphorylation of connexin-43, and normalized its distribution (not lateralized). Luzindole reversed all the above-mentioned effects. In conclusion, melatonin suppressed VF triggered by hypokalemia, prevented the action potential from widening, enhanced the electrical activity of the ventricles, and corrected connexin-43 misdistribution (431).

Diez et al. used both fructose-fed rats (FFR) and SHRs to assess the effects of melatonin on cardiac arrhythmia induced by IR injury (428). Both of these groups of rats exhibited abnormal metabolic features, including decreased ability of the myocardium to protect against oxidative stress, hypertension, reduced eNOS activity, etc. Melatonin suppressed the occurrence of VF in both groups and reduced VT severity. Melatonin affected both the length and amplitude of the action potentials, the length was shortened and the amplitude was restored. Therefore, the anti-arrhythmic effects of melatonin could be observed even in rats with genetic cardiac disease (428).

Table 6 lists some studies on the effects of melatonin on cardiac arrhythmias.

**Table 6:** Melatonin and cardiac arrhythmia.

**Melatonin dose**	**Treatment duration**	**Targets**	**Effects**	**Model**	**References**
10 mg/kg/d	7 days	SOD	Lowered VT, prevented VF occurrence	*In vitro, in vivo* (rats)	(427)
100 μM	–	Connexin-43	Suppressed VF triggered by hypokalemia, prevented action potential from widening, enhanced electrical activity of the ventricles	*In vivo* (rats)	(431)
50 μM	–	NADPH oxidase, eNOS	Protected against ventricular fibrillation when administered at reperfusion	*In vivo* (rats)	(428)
40 μg/mL	5 weeks	Cx43/PKC axis	Protected against lethal arrhythmias	*In vivo* (rats)	(426)
5, 10, 20, 50 μM	–	Total antioxidant capacity (TAC)	Reduced the incidence of reperfusion arrhythmia	*In vivo* (rats)	(284)
6 mg/kg/d	3 weeks	Lipid peroxides, nitrosative stress	Prevented ventricular arrhythmia	*In vivo* (hamsters)	(281)
10 μM	–	–	Prevented reperfusion-induced arrhythmia	*In vivo* (hamsters)	(432)
0.4 mg/kg	–	–	Lowered VF occurrence	*In vivo* (rats)	(433)
1/50,000 (v/v)	–	–	Reduced cardiac arrhythmia	*In vivo* (rats)	(434)

## Melatonin and Heart Failure

Heart failure (HF) is a complex clinical disease in the aging population characterized by the heart's inability to pump enough blood to the body as a result of structural and/or functional cardiac abnormalities (435). This syndrome is also associated with inadequate clearance of metabolic end products, leading to cardiovascular loss, followed by reparative fibrotic repair, ventricular remodeling, and eventually HF (39, 436). More than 37.7 million people worldwide are affected by HF, and this prevalence is rising (437). Importantly, despite recent advances in HF medical care and management, novel therapeutic approaches are required to reverse HF and restore heart tissue function. In this context, several experimental studies report the beneficial effects of melatonin treatment in variety HF models, including chronic intermittent hypoxia-induced HF, post-infarction HF caused by the left anterior descending coronary artery ligation, and isoproterenol-induced myocardial infarction (435, 438, 439). In these models, melatonin *via* its antioxidant properties cures significant pathogenic processes associated with HF including oxidative stress, apoptosis, necrosis, necroptosis, fibrosis, autophagy, inflammation, and pathological remodeling and dysfunction. Melatonin administration normalizes the blood pressure circadian rhythm, reduces cardiomyocyte loss, and improves the left ventricular function in patients with HF with reduced ejection fraction (HFrEF) (440). Melatonin is a molecule with a lot of electrons that may react with free radicals for directly scavenging them as well as up-regulating antioxidant enzymes. It acts as an antioxidant and protects cardiac tissue from ischemia and reperfusion injury caused by free O2 radicals and their byproducts. Melatonin also activates many antioxidative enzymes, such as glutathione peroxidase (GSH), modifies gene expression for various protective enzymes, and lowers lipid peroxidation (436). Melatonin reduces oxidative stress-induced cardiac tissue damage by increasing cardiac Na+/K+ ATPase and Sarco/endoplasmic reticulum calcium ATPase (SERCA) activity and decreasing myeloperoxidase (MPO) activity, caveolin-3, caspase-3 expression, and malondialdehyde (MDA) levels (441). Melatonin's protective impact has been related to its ability to increase antioxidant enzymes such as Cu/Zn superoxide dismutase, stimulate phosphorylated protein kinase B (p-Akt), and block caspase cascade activation, hence decreasing mesenchymal cell death (196, 442). Furthermore, the antioxidant effects of melatonin are most likely due to its inhibitory influence on NOS expression. Nitric oxide generates peroxynitrite and hydroxyl radicals, which cause membrane lipid peroxidation and oxidation of other molecules. Superoxide generation contributes to ventricular remodeling in heart failure, and as an antioxidant, melatonin appears to reduce myocardial remodeling (196).

Simko et al. in their study on rats with isoproterenol-induced heart failure found that melatonin declines the insoluble and total collagen and enhanced survival by modulating remodeling (443). Catecholamines and glucocorticoids, which are stress hormones, are also up-regulated in HF, boosting catabolic state and worsening cardiac failure. Melatonin is thought to lower catecholamine and cortisol levels in animal studies and may reverse their effects *via* antioxidant capabilities and activation of anabolic signaling pathways (444). Melatonin stimulates GH/IGF-1 signaling *via* the PI3k/AKT/mTOR pathway, activates AMPK pathway, and controls mitochondrial biogenesis to promote protein synthesis and reduce apoptosis.

Melatonin activates miR-200a-NF-E2-related factor 2 (Nrf2) in cardiomyocytes, causing adipose tissue to secrete C1q/tumor necrosis factor-related protein 3 (CTRP3). Because CTRP3 deficiency is linked to increased oxidative stress and cell death in cardiomyocytes, the influence of melatonin on CTRP3 secretion into the circulatory system and increased cardiac CTRP3 expression may help to prevent obesity in patients with heart failure with preserved ejection fraction (445). According to epidemiological research melatonin levels as a useful biomarker for HF reduced in patients with both acute and chronic HF. In this setting, serum melatonin levels have a negative correlation with N-terminal pro-brain natriuretic peptide (NT-pro-BNP), a well-known HF indicator (446). Surprisingly, it is also linked to reversal remodeling following cardiac resynchronization therapy in HF conditions (447). Melatonin affects homeostasis of extracellular matrix (ECM) in the left ventricular myocardium by improving the balance of MMP-1 and TIM-1 protein expression (445). Moreover, in ovariectomized rats with HF, melatonin prevents apoptosis and restores hypertrophy of contractile cardiomyocytes in the left ventricular myocardium. In one study decreased melatonin levels has been shown in patients admitted to hospital with congestive heart failure, so it is concluded that low melatonin levels exacerbate congestive heart failure (448).

**Figure 3:**
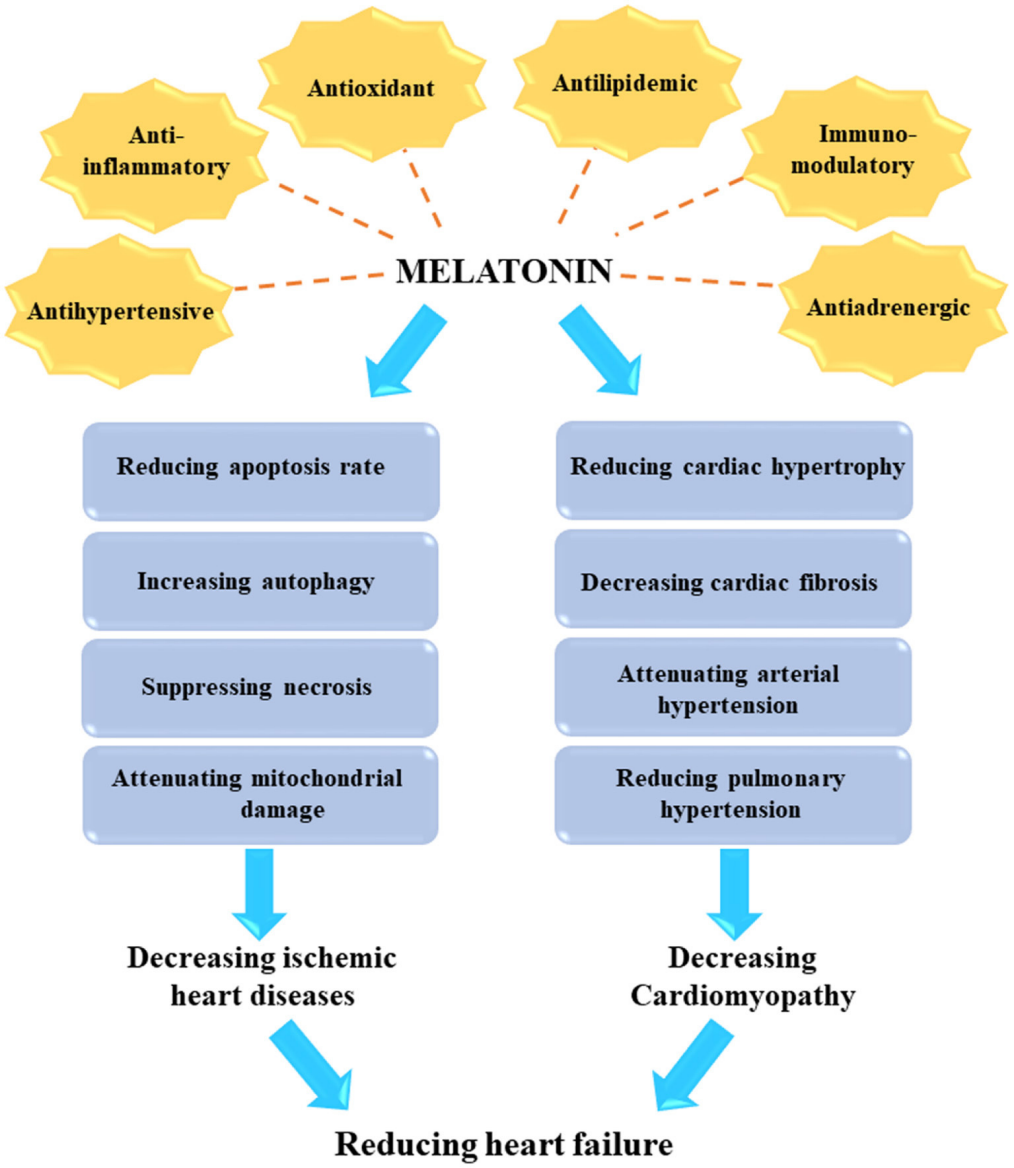
Beneficial effects of melatonin in reducing heart failure.

Melatonin's cytoprotective impact is dependent on the timing of delivery. Melatonin, when given early in the course of a myocardial infarction, has been shown to slow the progression of heart failure (444). Thanks to its antioxidant, anti-inflammatory and immunomodulatory properties, melatonin protects the heart against ischemic heart disease with myocardial cell death as well as post-infarction cardiac dysfunction and ischemic HF. Arterial hypertension and pulmonary hypertension induce both cardiac fibrosis and pathological regeneration (cardiomyopathy) with ventricular dysfunction and HF. Melatonin reverses these effects and prevents HF (435). Overall, melatonin seems to be an important, safe, and affordable molecule for the treatment of HF *via* improving cardiac defense mechanisms, which restores the myocardial function, muscle wasting, and cardiac cachexia. The beneficial effects of melatonin in HF have been illustrated in Figure 3.

## Melatonin as a Protective Agent Against Septic Cardiomyopathy

Approximately 60% of patients diagnosed with sepsis suffer the most common complication, namely septic cardiomyopathy. Elderly patients with septic cardiomyopathy have an estimated 30% risk of death, and the lack of effective therapies leads to a poor prognosis. Therefore, there is a need to for more research to clarify the pathogenesis of septic cardiomyopathy to improve the patient prognosis. Molecular studies have revealed that the pathogenesis of septic cardiomyopathy is related to endoplasmic reticulum (ER) stress and mitochondrial damage, because mitochondrial damage disturbs the energy supply of the myocardium, while ER stress inhibits protein turnover (266, 449). BAP31 (B-cell receptor-associated protein 31) regulates the apoptosis mediated by ER stress. Down-regulation of BAP31 suppresses cervical cancer development by altering the mitochondrial apoptosis pathway (450). BAP31 also affects apoptosis in colorectal cancer, and is linked to caspase-12. BAP31 regulates mitochondrial activity by interacting with TOMM40 (translocase of outer mitochondrial membrane 40), which explains the connections between the ER and mitochondria (451). BAP31 also transmits apoptosis signals from the ER to the mitochondria by interacting with CDIP1 (cell death-inducing p53-target protein 1) (452). Furthermore; BAP31 has been shown to be involved in Alzheimer's disease, hepatic steatosis, immunomodulation, and lipid metabolism (450–453). The MAPK/ERK axis increases cell survival through regulating genes connected to apoptosis. Pharmacological stimulation of the ERK pathway can reduce toxicity caused by lipopolysaccharide (LPS) in cardiomyocytes (453, 454). Furthermore, the ERK pathway appears to have a variety of effects on mitochondrial activity as well as ER stress. ERK influences ROS production in mitochondria, mitochondrial energy matabolism and membrane potential (455, 456). The ER has many functions in cells, including calcium homeostasis, protein synthesis, and the caspase-12 apoptotic pathway, which is controlled by the ERK pathway (457–459). Recent studies have revealed a close connection between BAP31 and the ERK pathway (455, 460).

Zhang et al. investigated the dual protective effects of melatonin on both mitochondria and ER, focusing on the BAP31 and ERK pathways. Their study investigated how BAP31 could affect the development of septic cardiomyopathy, and clarified the effect of melatonin on the ERK pathway, ER stress, and mitochondrial function (456). An *in vivo* septic cardiomyopathy model was created using lipopolysaccharide (LPS). Western blotting, quantitative-PCR and immunofluorescence were used to investigate the pathways. Due to the overwhelming inflammatory response and loss of cardiomyocytes, heart function was significantly disturbed after exposure to LPS. Melatonin treatment might increase cardiomyocyte survival by enhancing mitochondrial activity and decreasing ER stress. LPS suppressed BAP31 transcription, but melatonin restored BAP31 levels, which may be attributed to the MAPK-ERK axis. Inhibition of ERK and BAP31 blocked these effects. Overall, their study showed that melatonin affected the ERK-BAP31 axis to regulate ER-mitochondrial interactions during sepsis to prevent septic cardiomyopathy (456).

Irisin is a polypeptide hormone (112 amino acids) produced by proteolytic cleavage of the membrane bound FNDC5 (fibronectin type III domain-containing protein 5). Irisin and melatonin have both been investigated to see whether they can prevent the development of sepsis-related cardiac damage (457, 458). Irisin and melatonin have beneficial effects by control of ROS production, management of calcium homeostasis, and inhibition of cardiomyocyte apoptosis (458). Melatonin protects mitochondria in various conditions, including brain damage following cerebral hemorrhage, diabetic retinopathy, ARDS, IR injury in the liver, and endothelial cell oxidative dmage (461, 462). Irisin has protective effects on mitochondrial function in ischemia of the lung parenchyma, obesity, cardiac hypertrophy following hypertension, and cardiac IR injury (459, 463). According to numerous recent studies, Mst1 (mammalian STE20-like kinase 1) is a major subunit of the Hippo axis, which appears to be a primary regulator of mitochondrial homeostasis. High levels of Mst1 inhibited BCL2 activity and induced mitochondrial apoptosis. Furthermore, the posttranscriptional Mst1 levels directly regulate oxidative status and mitochondrial respiration (464). However, if the Mst1 level is low, mitochondrial autophagy would be activated to mediate the repair or elimination of mitochondria (464). Mst1 deletion also efficiently inhibited mitochondrial fission, and prevented the reduction in mitochondrial membrane potential and cardiomyocyte apoptosis (464). Despite the fact that Mst1 has been implicated in the regulation of mitochondrial homeostasis in numerous studies, it was unclear if it was involved in septic cardiomyopathy. JNK is a marker of myocardial damage since it is a downstream effector of Mst1. Increased JNK levels might promote Bax-mediated mitochondrial apoptosis by translocating it to the mitochondrial surface. Active JNK may also induce phosphorylation of Drp1 and c-Jun (465). Drp1 has been linked to mitochondrial fission, while c-Jun acts as a transcription factor for a number of genes that favor apoptosis (461, 466).

Ouyang et al. investigated whether melatonin combined with irisin could ameliorate septic cardiomyopathy by affecting the Mst1–JNK axis (462). According to their study, melatonin plus irisin decreased the depressive effect of LPS on the myocardium. LPS strongly activated Mst1, while the melatonin/irisin combination treatment substantially blocked this activation. Mst1 induced JNK activity, which increased oxidative damage, mitochondrial dysfunction, and apoptosis. The Mst1-JNK pathway was efficiently inhibited by melatonin plus irisin co-treatment, promoting myocardial viability and mitochondrial function. Upregulation of Mst-1 inhibited the beneficial effects of irisin and melatonin in the animal model, and also in cell culture, suggesting that irisin plus melatonin co-treatment inhibited septic cardiomyopathy by regulating the Mst1/JNK axis (462).

Autophagy is a process in which injured damaged proteins and dysfunctional organelles are degraded in lysosomes and recycled (463). The LC3-II/LC3-I ratio can measure autophagic activity. By binding to LC3-II, p62 is incorporated into autophagosomes and then destroyed during autophagy. As a result, p62 is negatively correlated with autophagy (467, 468). New studies have shown the involvement of autophagy in cardiac function, and have suggested that induction of autophagy could enhance heart function during sepsis (469, 470). On the other hand, a deficiency in autophagy exacerbated sepsis-induced heart failure (471). Recent research has shown that melatonin treatment could prevent the progress of cardiac dysfunction and reduce mortality and morbidity in sepsis (472, 473). SIRT1 is a histone deacetylase involved in many cellular processes, including senescence, inflammation, apoptosis, and autophagy (474–477). In a murine model of septic cardiac injury, upregulation of SIRT1 enhanced heart function (478). Melatonin has protective effects against cardiac dysfunction, and could also regulate SIRT1 activity in various diseases (206, 472, 473, 479, 480).

Zhang et al. examined the effects of melatonin on heart function in a mouse model of LPS-induced sepsis, and the involvement of SIRT1 (481). They assigned C57BL/6 mice into four groups: (1) control; (2) LPS; (3) LPS + melatonin; (4) LPS + melatonin + EX527 (SIRT1 inhibitor). The heart was examined for myocardial damage biomarkers, cardiac function, cardiomyocyte apoptosis, cardiac histology, autophagosomes, and expression of SIRT1, cleaved caspase-3, LC3-II/LC3-I ratio, and p62. When compared to the LPS group, melatonin reduced heart dysfunction, downregulated CK as well as CK-MB, decreased structural heart damage, prevented apoptosis, and promoted autophagy. Furthermore, melatonin also upregulated SIRT1 in cardiomyocytes, whereas EX527 inhibition of SIRT1 abrogated the cardioprotective effects of melatonin in sepsis. Melatonin rescued mice from septic heart damage by modulating apoptosis and autophagy, and activating SIRT1 (481).

Activation of Ripk3 is involved in the pathological development of inflammation (482). Ripk3 promoted mitochondrial dysfunction, which increased sepsis-induced kidney damage (483). This discovery was further reinforced by the finding that genetic deletion of Ripk3 decreased cardiomyocyte death by increasing mitochondrial homeostasis in a myocardial IR injury model (266). Furthermore, Ripk3 activity and ER stress have been observed to be connected in several diseases (484). Ripk3 appears to be able to reduce damage to mitochondria as well as ER stress (485, 486), and could also affect sepsis-induced cardiac injury. Tryptophan and melatonin both affect the immune system, cell apoptosis, and microvascular vasoconstriction in numerous disease models, including heart IR injury, hepatic steatosis, acute renal injury (ARI), Parkinson's disease (PD), and cancer (14, 232, 487–489). Melatonin has also been extensively studied for the treatment of sepsis, and many protective mechanisms have been postulated. These have included, inflammasome inhibition, PI3K/Akt pathway activation, and antioxidant activity. Researchers reported that melatonin inhibited the activation of Ripk3, and had pro-survival effects in the reperfused heart (266). However, the effect of melatonin on Ripk3 in septic cardiomyopathy had not been shown.

One study explored how Ripk3 levels affected cardiac dysfunction induced by sepsis, and whether melatonin could improve it and the molecular mechanism (458). Following induction of inflammation in the myocardium by LPS, Ripk3 was elevated in heart tissue samples, along with cardiomyocyte death. Septic myocardial damage was reduced after treatment with melatonin, which had the same effect as genetic deletion of Ripk3. Molecular studies have shown that Ripk3 can regulate mitochondrial activity, ER stress, and cytoskeleton organization, resulting in a cardioprotective activity. To summarize, melatonin-mediated inhibition of Ripk3 in cardiomyocytes enhanced bioenergetics and decreased oxidative damage in the mitochondria. Melatonin also alleviated the effects of ER stress in cells and restored calcium recycling. When LPS-treated hearts were infected with an adenovirus expressing Ripk3, melatonin treatment became ineffective due to the overexpression of Ripk3. It was concluded that Ripk3 overexpression was associated with septic cardiomyopathy, which could be suppressed by melatonin, presumably by inhibiting Ripk3 (458).

Table 7 lists some studies on the effects of melatonin on septic cardiomyopathy.

**Table 7:** Effects of melatonin on septic cardiomyopathy.

**Melatonin dose**	**Route of administration**	**Effects**	**Model**	**References**
20 mg/kg	Intraperitoneal	Suppressed septic cardiac injury, by regulating mitochondrial and ER activity, cytoskeletal organization	*In vivo* (mice)	(458)
30 mg/kg	Intraperitoneal	Mitigated septic cardiac injury *via* activation of PI3K/Akt signaling	*In vivo* (rats)	(472)
30 mg/kg	Intraperitoneal	Prevented sepsis-dependent mitochondrial injury, improved mitochondrial respiration	*In vivo* (mice)	(490)
30 mg/kg	Intraperitoneal & subcutaneous	Suppressed iNOS/imtNOS activity triggered by sepsis, restored mitochondrial function	*In vivo* (mice)	(491)
30 mg/kg	Intraperitoneal & subcutaneous	Inhibited iNOS/imtNOS activity, enhanced mitochondrial function, & nNOS/c-mtNOS	*In vivo* (mice)	(492)
30 mg/kg	Intraperitoneal	Regulated autophagy & apoptosis through modifying UCP2	*In vivo* (mice)	(473)
100 nM	–	Regulated autophagy & apoptosis through modifying UCP2	*In vitro*	(473)
30 mg/kg	Intraperitoneal & subcutaneous	Reduced NLRP3 level & activity, inhibited caspase-1 and IL-1β	*In vivo* (mice)	(493)
30 mg/kg	Intraperitoneal & subcutaneous	Upregulated cytochrome c oxidase, promoted systolic cardiac activity, reduced mortality	*In vivo* (rats)	(494)
30 mg/kg	Intraperitoneal	Activated SIRT1, regulated apoptosis & autophagy, suppressed septic cardiomyopathy	*In vivo* (mice)	(481)
10 mg/kg	Intraperitoneal	Prevented organ damage, free radical scavenger, & antioxidant activity	*In vivo* (rats)	(495)
10, 20 mg/kg 10–20 μM	Intraperitoneal	Stabilized BAP31 *via* ERK pathway, preserved cardiac function in septic cardiomyopathy	*In vivo* (mice) *In vitro*	(456)

## Clinical Trials Using Melatonin for Cardiovascular Diseases

The experimental studies led to clinical trials, in which melatonin was administered in combination with heart disease medications for the prevention and treatment of CVDs.

According to an analysis of the results of numerous clinical trials, melatonin is useful in decreasing nocturnal hypertension when supplied in a controlled-release form, but useless when administered as a fast-release formulation. A patent issued in the United States in December 2011 describes a formulation for the prevention and treatment of hypertension symptoms in people who are resistant to classical hypertension (496). This formula contains an antihypertensive drug (e.g., captopril, diltiazem, etc.) and melatonin, which is expected to lower blood pressure, especially nocturnal hypertension, in patients with nocturnal hypotension. Treatment with this combination can also reduce cortisol production and postpone serum cortisol levels, thereby lowering the risk of morning ischemic attacks. The compound is prescribed in a controlled-release formulation because the patent reports that fast-acting or controlled-release melatonin has different effects on blood pressure, cortisol levels, and mood of patient. In particular, melatonin with controlled-release lowers diastolic and systolic blood pressure throughout the day without having a significant effect on patients with normal blood pressure, but regular formulation of melatonin (5 mg) has been observed to lower blood pressure in individual with normal blood pressure. This patent does not provide any information about the co-administration of melatonin and an antihypertensive drug (496).

Ahsanova et al. have lunched a study to assess the efficacy and safety of melatonin therapy in patients with hypertension based on the evaluation of daily blood pressure monitoring (497). They reported that after melatonin intake, average 24 h brachial systolic (SBP) and diastolic blood pressure (DBP)values decreased significantly from 124.6 ± 12.1 to 121.0 ± 10.2 mmHg and 79.7 ± 8.8 to 77.3 ± 6.5 mmHg, respectively, as well as average day-time SBP and DBP values from 128.2 ± 13.2 to 122.5 ± 9.9 and 82.3 ± 9.7 to 78.5 ± 7.2 mmHg, considerably (497).

In another study, melatonin 5 mg reduced nocturnal blood pressure but increased daytime blood pressure in non-dippers with coronary artery disease (363). However, melatonin 24 mg sustained-release had no effect on nocturnal blood pressure in African Americans with essential hypertension (NCT01114373) (347). Melatonin administration prior to coronary artery bypass grafting increased EF, decreased heart rate, and lowered markers of reperfusion injury in a dose-dependent manner (10 vs. 20 mg, 5 days before surgery), and in another study, 10 mg melatonin administration for 1 month prior to coronary artery bypass grafting increased antioxidant defense. Likewise, 12 weeks of 10 mg melatonin treatment had a positive effect on antioxidant capacity, glycemic management, HDL cholesterol, and blood pressure in diabetic patients with coronary heart disease (348). One study has been conducted in order to compare the efficacy of melatonin monotherapy (MT) and combined treatment (CT) in elderly patients (mean age 64 years) with arterial hypertension (AH) and coronary heart disease (CHD) (498). The results indicate that MT has an antihypertensive effect. In the control groups, CT with melatonin and antihypertensive medications outperformed standard therapy. Melatonin inclusion in CT of CHD produced significant anti-ischemic and anti-anginal benefits, as well as corrected oxidant/antioxidant balance (498). Therefore, melatonin should be considered as an important element of MT and CT of cardiovascular disorders. In recently published paper, evaluation of the effect of melatonin on endothelial function in HF explored that oral melatonin for 24 weeks had a beneficial effect on endothelial function in patients with HF with reduced ejection fraction (HFrEF) (499).

Melatonin supplementation (20 mg for 8 weeks) has been shown in a randomized controlled trial to improve fatigue, appetite, and quality of life in HF patients with cachexia, and the combination of melatonin and branched chain amino acids amplifies these effects (500).

## Conclusions

The study of the pineal gland, and the effects of melatonin on a wide range of organs and bodily systems is a growing field of research. It has long been known that the cardiovascular system is affected by circadian rhythms, and this connection has now been realized to have therapeutic significance. Melatonin is a ubiquitous component of the human diet, and is also widely available as a healthfood supplement. While many of the applications of melatonin supplements have been directed to normalizing sleep patterns, treating jet-lag and insomnia, the benefits of melatonin for other conditions are becoming increasingly understood. It is well-known that melatonin is an antioxidant and free redical quencher, but the discovery of high affinity receptors for melatonin expressed in many tissues has widened the scope of mechanistic investigations. Nevertheless, although research into the role of melatonin in CVDs has only recently begun in earnest, the literature on this subject has been expanding to such an extent, that it is difficult to summarize in a single article. Nearly all the studies have reported positive effects of melatonin on cardiovascular physiology, and the prevention of damage to the myocardium after heart attack, IR injury, or sepsis. Melatonin can also help blood pressure and heart arrythmia. Since melatonin is inexpensive and non-toxic if consumed in reasonable quantities, it should be tested in many more extensive clinical trials to assess its efficacy in a variety of cardiovascular disorders. Moreover, some clinical studies indicated that utilization melatonin in CVDs is associated with more inconsistencies regarding its cardioprotective effects (501, 502). “Apart from dosage issues and mode of administration, previous failures could be partially explained by the use of young and healthy animals with eventual lack of various cardiovascular risk factors, comorbidities and comedications which are characteristics of patients suffering an acute myocardial infarction or undergoing cardiovascular surgery (503). Considering the current disappointment, further well-planned preclinical and clinical studies are needed to better delineate the cardiovascular benefits of melatonin” (15).

## Author Contributions

MT, SM, FD, AA, HK, MH, ZA, RJR, and HM contributed in conception, design, and drafting of the manuscript. All authors contributed to the article and approved the submitted version.

## Conflict of Interest

The authors declare that the research was conducted in the absence of any commercial or financial relationships that could be construed as a potential conflict of interest.

## Publisher's Note

All claims expressed in this article are solely those of the authors and do not necessarily represent those of their affiliated organizations, or those of the publisher, the editors and the reviewers. Any product that may be evaluated in this article, or claim that may be made by its manufacturer, is not guaranteed or endorsed by the publisher.
